# Analysis of Acyl Solamines in Tuber Periderm of Cultivated and Wild Potatoes Using Liquid Chromatography Coupled With Electrospray Ionization Quadrupole Time‐of‐Flight Mass Spectrometry

**DOI:** 10.1002/jms.5177

**Published:** 2025-09-21

**Authors:** Christoph Böttcher, Karin Gorzolka, Paul Himmighofen, Torsten Meiners

**Affiliations:** ^1^ Julius Kühn‐Institute, Federal Research Centre for Cultivated Plants Institute for Ecological Chemistry, Plant Analysis and Stored Product Protection Berlin Germany

**Keywords:** alkaloids, all‐ion fragmentation, *Solanum cardiophyllum*, *Solanum pinnatisectum*, *Solanum tuberosum*, tandem mass spectrometry

## Abstract

Acyl solamines are a poorly investigated class of alkaloids found in several solanaceous plants. A general screening approach on the basis of all‐ion fragmentation is proposed for this compound class using reversed‐phase ultra‐high performance liquid chromatography coupled with electrospray ionization quadrupole time‐of‐flight mass spectrometry. When applied to tuber periderm extracts of *Solanum tuberosum* , *Solanum pinnatisectum* , and *Solanum cardiophyllum* , this approach resulted in the annotation of over 20 acyl solamines including short‐ and medium‐chain fatty acyl solamines, hydroxycinnamoyl solamines, and other aromatic acyl solamines. In addition, minor derivatives with *N*‐oxidized solamine moieties (acyl solamine‐*N*‐oxides and di‐*N*‐oxides) and *N*‐demethylated solamine moieties (acyl nor‐ and dinor‐solamines) were detected. The annotated compounds formed singly and doubly protonated molecules under positive ion electrospray conditions. Upon collision‐induced dissociation (CID), protonated acyl solamines and derivatives with modified solamine moieties produced informative and readily interpretable product ion spectra. This enabled the structural characterization of the solamine head group and, in some cases, of the acyl moiety. To localize the positions of the carbon–carbon double bonds, major unsaturated medium‐chain fatty acyl solamines were isolated in small quantities from *S. tuberosum* tuber periderm and derivatized with meta‐chloroperbenzoic acid. Analysis of the CID mass spectra obtained from the protonated oxidation products provided valuable information for the further structural characterization of these compounds.

## 1 Introduction

Solapalmitine (*N*,*N*‐bis‐(4‐dimethylaminobutyl)‐hexadecanamide, C16:0‐solamine) and solapalmitenine (*N*,*N*‐bis‐(4‐dimethylaminobutyl)‐hexadec‐2‐enamide, C16:1(2)‐solamine) were the first discovered representatives of long‐chain fatty acyl (LCFA) solamines. Both alkaloids were isolated from the aerial parts of *Solanum tripartitum* almost six decades ago [1, 2]. Solapalmitine exhibited significant cytotoxicity and antitumor activity [3], which is related to its ability to alter the permeability of cell membranes [4, 5]. Further solamine conjugates were isolated from the roots of *Solanum betaceum* and *Solanum carolinense* , including the medium‐chain fatty acyl (MCFA) solamine solacaproin (C6:0‐solamine) and the urethane derivative solalaurethine (*N*‐ethoxycarbonyl‐solamine), respectively [6, 7]. Thin‐layer chromatographic analyses of the roots and aerial parts of numerous *Solanum* species indicated a broader occurrence of solamine and acyl solamines within this genus [8]. Recently, LC/MS‐based metabolite profiling of leaf tissue from an accession of the wild potato species *Solanum bulbocastanum* , which is resistant to the Colorado potato beetle ( *Leptinotarsa decemlineata* ) and *Phytophthora infestans* , the causal agent of late blight, revealed the presence of a multitude of LCFA solamines [9]. The most abundant representative, which accumulates at concentrations of up to 1.8% of leaf dry matter, was isolated and identified as (7*Z*,10*Z*,13*Z*)‐hexadecatrienoyl solamine. The isolated LCFA solamine exhibited anti‐feeding activity against the Colorado potato beetle and anti‐oomycete activity against *P. infestans* sporangia. Further targeted LC/MS analyses using an *in silico* generated compound database revealed that numerous wild potato species, as well as table potato cultivars, accumulate acyl solamines particularly in belowground organs in a species‐ and organ‐specific manner [9].

Given the occurrence of acyl solamines in both wild and cultivated potatoes and the interesting bioactivities of the known LCFA solamines and their hypothesized role in plant defense, this manuscript presents a rapid nontargeted screening approach for these alkaloids using ultra‐high performance liquid chromatography coupled with electrospray ionization quadrupole time‐of‐flight mass spectrometry (UHPLC/ESI‐QTOFMS) and all‐ion fragmentation. The approach is applied to comprehensively analyze the profile of acyl solamines and related derivatives in the tuber periderm of a table potato cultivar and of two wild potato species of the series *Pinnatisecta*. Compared with the recently described targeted screening approach [9], the developed non‐targeted screening approach revealed the presence of numerous novel minor acyl solamine derivatives that had previously remained undiscovered. The manuscript provides a collection of chromatographic and collision‐induced dissociation (CID) tandem mass spectral data of the detected acyl solamines and minor derivatives with modified solamine head group as well as a discussion of their fragmentation behavior. In addition, the positions of the C‐C double bonds in three major unsaturated MCFA solamines accumulating in the tuber periderm of a table potato cultivar were partially determined based on the interpretation of the CID mass spectra obtained from their protonated epoxidation products.

## 2 Material and Methods

### 2.1 Chemicals

Methanol (≥ 99.95%, for LC–MS grade), acetonitrile (≥ 99.95%, for LC–MS), and dichloromethane (≥ 99.9%, for residue analysis) were purchased from Th. Geyer GmbH & Co. KG. Ultra‐pure water (resistivity ≥ 18.2 MΩ cm) was obtained from a water purification system (AriumPro, Sartorius AG). Formic acid (≥ 99.0%, LC–MS grade) was purchased from Carl Roth GmbH + Co. KG, acetic acid (≥ 99.9%, LC–MS grade) from Biosolve, and meta‐chloroperbenzoic acid (m‐CPBA, ≤ 77%) from Sigma‐Aldrich.

C16:0‐solamine dihydrochloride was obtained from the reaction of solamine (4,4′‐bis (dimethylamino)dibutylamine) with palmitoyl chloride (98%, Sigma‐Aldrich) in the presence of pyridine. C10:1(2*E*)‐solamine dihydrochloride and C10:1(2*Z*)‐solamine dihydrochloride were prepared from solamine and (2*E*)‐decenoic acid (≥ 95.0%, TCI) and (2*Z*)‐decenoic acid (≥ 95.0%, Sigma‐Aldrich) using *O*‐(benzotriazol‐1‐yl)‐*N*,*N*,*N*′,*N*′‐tetramethyluronium hexafluorophosphate (Sigma‐Aldrich) as a coupling reagent and *N*,*N*‐diisopropylethylamine as a base. Solamine itself was synthesized in a four‐step sequence starting from 4‐(*tert*‐butyloxycarbonylamino)butanoic acid and *tert*‐butyl *N*‐(4‐aminobutyl)carbamate. Detailed synthetic procedures will be reported in a separate publication. NMR data of the synthesized fatty acyl solamine dihydrochlorides are given in Supporting Information.

### 2.2 Plant Material

*Solanum pinnatisectum* Dunal (clonal line pnt2G, accession number WKS 31607, IPK Gene Bank, Gatersleben, Germany) and *Solanum cardiophyllum* Lindl. (clonal line cph 6/4, accession number WKS 30086, IPK Gene Bank) were regenerated from callus cultures [10] and kindly provided as 3‐week‐old plantlets by Dr. Roman Gäbelein (Julius Kühn‐Institute, Sanitz, Germany). Plantlets were transferred to potting soil and grown in a climate chamber under long‐day conditions as described [9]. *Solanum tuberosum* ‘Quarta’ was grown from seed tubers in potting soil under the same conditions. Tubers were harvested after 12 to 14 weeks and gently cleaned with tap and deionized water. Large tubers were cut into 1‐cm‐thick slices. Immediately after harvest, tuber samples were frozen in liquid nitrogen and freeze‐dried for 7 days (Gamma 1–16 LSC, condenser temperature −50°C, pressure 0.52 mbar, Martin Christ Gefriertrocknungsanlagen GmbH). After drying, the tuber periderm was separated from the underlying tuber phelloderm using tweezers. Tuber periderm samples were transferred into 2‐mL microcentrifuge tubes and ground (5 min, 30 Hz) into a fine powder using a steel bead (diameter 8 mm) and a mixer mill (MM300, Retsch GmbH). For each sample, periderm isolated from tubers of three individual plants was pooled.

### 2.3 Extraction

Homogenized tuber periderm (10 mg) was precisely weighed into a 2‐mL microcentrifuge tube and methanol/water, 4/1 (v/v) (125 μL per mg dry weight) and two steel beads (diameter 4 mm) were added. The mixture was vigorously shaken (5 min, 30 Hz, room temperature) using a mixer mill (MM300). After removal of the steel beads, the mixture was sonicated (5 min, room temperature), shaken (30 min, 1800 min^−1^, room temperature) and centrifuged (10 min, 13 000 × g, room temperature). An aliquot of the supernatant was 10‐fold serially diluted with methanol/water, 4/1 (v/v). Undiluted and diluted (1/10, 1/100) extracts were subjected to LC/MS analysis.

### 2.4 Quantification of Acyl Solamines

Matrix matched calibration samples (20 μM, 5 μM, 2 μM, 500 nM, 200 nM, 50 nM, 20 nM, and 5 nM) were prepared using a stock solution (2 mM) of C16:0‐solamine dihydrochloride in methanol and a *S. tuberosum* ‘Quarta’ tuber periderm extract, which was 1/10 diluted with methanol/water, 4/1 (v/v). Calibration samples were analyzed in duplicate before and after the tuber periderm extracts (bracketed calibration). For quantification of acyl solamines extracted ion chromatograms of [M + H]^+^ (*m/z*‐width ±20 ppm) were integrated. Peak integrals of isomeric compounds **11**, **13**, and **17–20** were summed up. An external calibration curve (quantifier ion: *m/z* 454.473 [M + H]^+^, t_R_ 10.37 min, calibration range: 0.02–2 μM, 5 points in duplicate, regression model: y = ax^b^, 1/x^2^ weighted, R^2^ = 0.9954) was established for C16:0‐solamine and used for semiquantification of other acyl solamines. If the peak integral of an acyl solamine in the undiluted tuber periderm extract exceeded the calibration range, 1/10 or 1/100 diluted tuber periderm extracts were used for quantification.

### 2.5 UHPLC/ESI‐QTOFMS

LC/MS analyses were performed on an Infinity 1290 series UHPLC system (Agilent Technologies) consisting of a binary pump (G4220A), an autosampler (G4226A, 20 μL loop), an autosampler thermostat (G1330B), a thermostatted column compartment (G1316C), which was coupled in series with a diode array detector (G4212B) and an iFunnel Q‐TOF mass spectrometer (G6550A, Agilent Technologies) via a dual Agilent jet stream electrospray ion source. MassHunter LC/MS Data Acquisition software (version B.06.01) was used for controlling the instrument and data acquisition as well as MassHunter Qualitative and Quantitative Analysis software (version B.07.00) for data evaluation. The mass spectrometer was operated in low mass range (*m/z* 1700) and extended dynamic range (2 GHz) mode. Using these settings, the mass resolution (full width at half maximum) at *m/z* 922 was approx. 23,000. The instrument was auto‐tuned and calibrated according to the manufacturer's recommendations using ESI‐L tuning mix (Agilent Technologies).

Extracts (1 μL injection volume) were separated on an ACQUITY UPLC HSS T3 column (2.1 mm × 100 mm, particle size 1.8 μm, pore size 100 Å, Waters) using 0.5% (v/v) formic acid in water and 0.5% (v/v) formic acid in acetonitrile as eluent A and B, respectively. The following binary gradient program at a flow rate of 500 μL min^−1^ was applied: 0 to 1 min: isocratic, 5% B; 1 to 15 min: linear from 5% to 75% B; 15 to 18 min: isocratic, 95% B; 18 to 20 min: isocratic, 5% B. The column temperature was maintained at 40°C and the autosampler temperature at 6°C. Absorption spectra were acquired in a wavelength range of 190 to 600 nm using an acquisition rate of 2.5 spectra per second. Mass spectra were acquired in positive ion mode from *m/z* 70 to 1700 using an acquisition rate of 3 spectra per second. The following instrument settings were applied: nebulizer gas: nitrogen, 35 psig; dry gas: nitrogen, 200°C, 18 L min^−1^; sheath gas: nitrogen, 300°C, 12 L min^−1^; capillary voltage: 3000 V; nozzle voltage: 0 V; high pressure funnel: voltage drop 200 V, RF voltage 150 V; low pressure funnel: voltage drop 100 V, RF voltage 100 V; funnel exit DC voltage: 50 V; octopole RF voltage: 750 V; collision gas: nitrogen; collision energy: 0 V. Centroid mass spectra were acquired. For all‐ion fragmentation experiments, collision energy was alternated between 0 and 25 V.

CID mass spectra were acquired in targeted‐MS/MS mode using scheduled precursor ion lists and the following parameters: acquisition rate MS: 3 spectra per second; acquisition rate MS/MS: 4 spectra per second, isolation width: narrow (1.3 *m/z*); collision energy: 10, 25, 40 V; collision gas: nitrogen. For acquisition of CID mass spectra of in‐source fragment ions (pseudo‐MS^3^) funnel exit DC voltage was increased from 50 to 100 V.

Reference mass correction was used for all analyses and experiments. For this purpose, a solution of purine (35 μM, Sigma‐Aldrich) and hexakis‐(2,2,3,3‐tetrafluoropropoxy)phosphazine (35 μM, Apollo Scientific) in acetonitrile/water, 95/5 (v/v) was continuously introduced through the second sprayer of the dual ion source at a flow rate of 18 μL min^−1^ using an external HPLC pump equipped with a 1:100 splitting device.

### 2.6 Isolation of Acyl Solamines From Tuber Periderm

Field‐grown *S. tuberosum* ‘Quarta’ tubers were washed with tap water to remove adhering soil. After drying, tubers were peeled using a vegetable peeler. The obtained peels were frozen at −80°C in a freezer, freeze‐dried, and homogenized using a tube mill (IKA Werke GmbH & Co. KG). A mixture of 20 g tuber peel homogenate and 200 mL methanol/water, 4/1 (v/v) was sonicated for 10 min (bath temperature ≤ 35°C). Afterwards, the mixture was vigorously stirred for 2 h at room temperature using a magnetic stirrer and vacuum filtered using a Büchner funnel. The filter cake was extracted once again with 200 mL methanol/water, 4/1 (v/v) following the above‐described procedure. The obtained raw extracts were combined, acidified with 2 M HCl to pH 2, and subjected to solid‐phase extraction using two cartridges (60 mL) packed with polymeric Strata‐X‐C sorbent (8 g, particle size 33 μm, Phenomenex). The cartridges were conditioned with 100 mL methanol and equilibrated with 100 mL methanol/water, 4/1 (v/v). The acidified raw extract was then applied in equal parts to the cartridges, which were subsequently washed with 100 mL methanol/0.5 M hydrochloric acid, 4/1 (v/v) and 100 mL methanol. Basic compounds were eluted with 100 mL methanol/25% aqueous ammonia, 95/5 (v/v). Eluates from both cartridges were combined and evaporated to dryness *in vacuo* using a rotary evaporator (bath temperature 35°C) to give 221 mg of a brownish oil. An aliquot (60 mg) of this oily residue was solubilized in 1500 μL methanol/water, 4/1, and separated using an AZURA preparative HPLC system (KNAUER) equipped with an Atlantis T3 OBD Prep column (19 mm × 250 mm, particle size 5 μm, Waters). Water and acetonitrile both acidified with 0.5% (v/v) formic acid were used as eluent A and B, respectively. The following binary gradient program at a flow rate of 20 mL min^−1^ was applied: 0–15 min: linear from 5% to 35% B; 15–15.5 min: linear from 35% to 95% B; 15.5–21.5 min: isocratic, 95% B. An injection volume of 300 μL was used. Fractions were collected from 9–15 min every 30 s. Collected fractions were subjected to UHPLC/ESI‐QTOFMS and screened for target compounds. Fractions containing **12a** (t_R_ 11.0–11.5 min) and **10a** and **12b** (t_R_ 13.0–13.5 min), which were not separable, were evaporated to dryness *in vacuo* using a rotary evaporator. The remaining residues were dissolved in methanol (500 μg/mL) and stored in the freezer at −20°C until further use.

### 2.7 Oxidation of Isolated Fatty Acyl Solamines With Meta‐Chloroperbenzoic Acid

An aliquot (100 μL) of the fatty acyl solamine containing fraction (500 μg/mL) was transferred into a 2‐mL microcentrifuge tube. After removal of the solvent using a vacuum centrifuge, 50 μL of a freshly prepared solution of m‐CPBA in dichloromethane (10 mg/mL) were added. The mixture was briefly vortexed and allowed to stand at room temperature for 10 min. After addition of 950 μL acetonitrile/water, 3/1 (v/v) the mixture was again briefly vortexed and subjected to UHPLC/ESI‐QTOFMS. CID tandem mass spectra of the protonated oxidation products were acquired in targeted‐MS/MS mode as described above.

## 3 Results and Discussion

### 3.1 Optimization of the Chromatographic Separation of Acyl Solamines

Chromatographic separation of methanolic tuber periderm extracts under the conditions routinely used in our laboratory for LC/MS‐based profiling of semipolar plant metabolites [11] produced broad and tailed peaks for acyl solamines. In order to improve their chromatographic separation, the effect of the stationary phase, the eluent additive, the organic modifier, and the column temperature on the retention time, peak width, and tailing of the model compound C16:0‐solamine was studied (Table S1). In all experiments conducted, retention and peak shape of C16:0‐solamine were slightly better on the Acquity UPLC HSS T3 phase than on the Zorbax RRHD Eclipse Plus C18 phase. Gradual improvement of the peak shape was achieved by stepwise increasing the concentration of formic acid from 0.1% to 0.5%, the maximum tolerable concentration with respect to column stability and corrosiveness. Using acetic acid (0.5%) instead of formic acid (0.5%) as eluent additive proved counterproductive. Trifluoroacetic acid was not tested as eluent additive due to its signal‐suppressing effects in electrospray ionization mass spectrometry [12]. However, it can be hypothesized that its higher acidity and capacity for ion pairing compared with formic acid may positively influence the retention and peak shape of acyl solamines [13]. Significant increases in the retention time and the peak width of C16:0‐solamine were observed when the organic modifier acetonitrile was substituted with methanol. In contrast, elevating the column temperature from 40°C to 50°C resulted in minimal alterations in retention time and peak shape. Based on these findings, a generic chromatographic method with a cycle time of 20 min was developed, enabling rapid gradient separation of diverse acyl solamine classes.

### 3.2 Screening for Acyl Solamines and Related Derivatives Using All‐Ion Fragmentation

**FIGURE 1:**
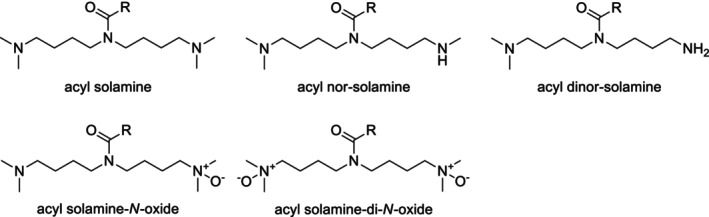
General structures of acyl solamines and derivatives with modified solamine head group.

Upon CID, protonated acyl solamines and related derivatives including acyl nor‐solamines, dinor‐solamines, and solamine‐*N*‐oxides (Figure 1) form a series of common product ions. Among them, the product ion at *m/z* 100.112 (Me_2_Py^+^, C_6_H_14_N^+^) is the most abundant one at medium collision energies (CEs) in the range of 20 to 30 V. This ion can therefore be readily used as an indicator product ion to sensitively screen for acyl solamines and related derivatives using UHPLC/ESI‐QTOFMS in combination with all‐ion fragmentation (AIF). In this data‐independent data acquisition approach, mass spectra are alternately recorded at low and high CE without prior precursor ion selection [14].

To identify acyl solamines and related derivatives from AIF LC/MS raw data, narrow mass‐window extracted ion chromatograms generated for *m/z* 100.112 (±0.002) from high‐CE (25 V) mass spectra are evaluated. At the apexes of the detected peaks, adjacent low‐CE (0 V) mass spectra are inspected in order to identify the associated precursor ions. This step is facilitated by the characteristic ionization behavior of acyl solamines and related derivatives, which form both singly and doubly protonated molecules upon electrospray ionization. Based on the accurate *m/z* and relative isotope abundances of the protonated molecule, the elemental composition of the putative acyl solamine or related derivative is determined. To avoid structural misannotations, in particular of species that are low in abundance and elute in crowded chromatographic regions, it is advisable to acquire CID tandem mass spectra of the protonated acyl solamine derivatives in targeted‐MS/MS mode using a scheduled precursor ion list. Alternatively, CID mass spectra of well‐separated, abundant acyl solamines can be extracted directly from the high‐CE mass spectra of the AIF LC/MS raw data. Based on the elemental composition and the CID mass spectrum, the type of the solamine head group and the elemental composition of the acyl moiety are determined for individual acyl solamine derivatives. It should be noted that protonated acyl solamine‐di‐*N*‐oxides (Figure 1) do not produce abundant indicator product ions at *m/z* 100.112 upon CID. To detect acyl solamines and all derivatives with modified head groups as shown in Figure 1, the common product ion at *m/z* 126.128 (e^+^, C_8_H_16_N^+^) can be used as an alternative indicator product ion. However, due to its generally lower relative intensity (~10% at CE = 25 V) compared with the product ion at *m/z* 100.112 (base peak at CE = 25 V), sensitivity is reduced by an order of magnitude here.

### 3.3 Acyl Solamines and Related Derivatives in Tuber Periderm of Cultivated and Wild Potatoes

**FIGURE 2:**
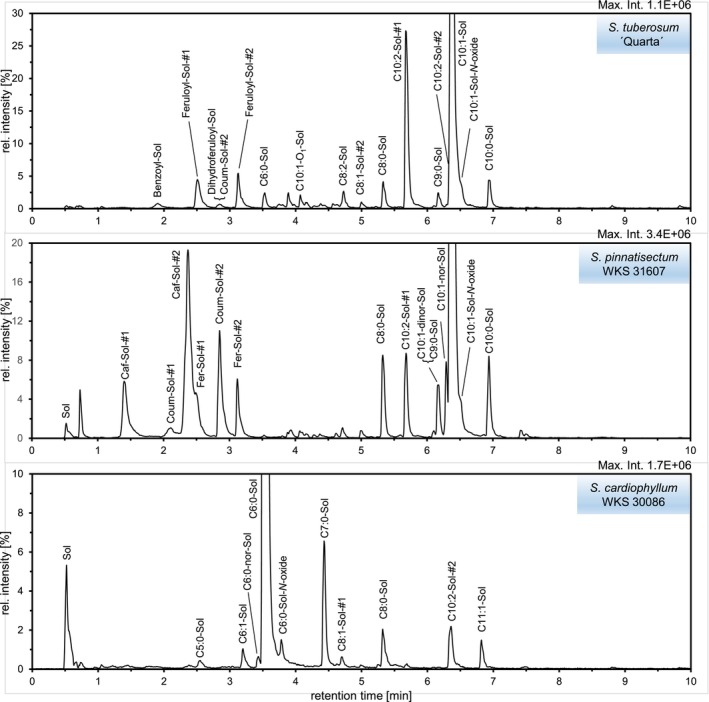
Detection of acyl solamines in tuber periderm extracts of cultivated and wild potatoes by UHPLC/ESI(+)‐QTOFMS operated in all‐ion fragmentation mode. Extracted ion chromatograms (maximum intensity scaled to 100%) of the dominant common product ion Me_2_Py^+^ at *m/z* 100.112 ± 0.002 are shown. Chromatograms were obtained at a collision energy of 25 V using 1 μL of a diluted tuber skin extract (concentration 0.8 mg dry mass mL^−1^).

To demonstrate the applicability of the proposed screening approach, methanolic extracts were prepared from freeze‐dried tuber periderm of a table potato cultivar ( *S. tuberosum* ‘Quarta’) and individual gene bank accessions of two wild potato species ( *S. pinnatisectum* WKS 31607, *S. cardiophyllum* WKS 30086) and analyzed by AIF LC/MS. The corresponding extracted ion chromatograms of the indicator product ion at *m/z* 100.112 are shown in Figure 2. The analysis of these chromatograms in conjunction with the evaluation of separately acquired CID tandem mass spectra led to the annotation of one short‐chain fatty acyl solamine, 15 MCFA solamines, three hydroxycinnamoyl (HCA) solamines, and three additional acyl solamines with aromatic acyl moieties. The annotated derivatives with a modified head group include two MCFA nor‐solamines, three HCA nor‐solamines, one MCFA dinor‐solamine, two MCFA solamine‐*N*‐oxides, and two MCFA solamine‐di‐*N*‐oxides. The elemental compositions, retention times, and *m/z* values of singly and doubly protonated molecules of all annotated compounds are given in Table 1. The nomenclature of the fatty acyl moieties follows the proposed shorthand notation for MS‐derived lipid structures [15].

**TABLE 1:** Elemental compositions, analytical data and levels of acyl solamines and derivatives detected in the tuber periderm of *S. tuberosum* ‘Quarta’, *S. pinnatisectum* WKS 31607, and *S. cardiophyllum* WKS 30086.

No.	Name	Elemental composition	t_R_ (min)	*m/z* [M + H]^+^ (error in ppm)	*m/z* [M + 2H]^2+^ (error in ppm)	Level^a^ [nmol (g dry matter)^−1^]		
						*S. tub*. ‘Quarta’	*S. pinnat*. WKS 31607	*S. cardio*. WKS 30086
**1**	C5:0‐solamine^b^	C_17_H_37_N_3_O	2.53	300.3011 (0.4)	150.6544 (2.1)	3	n.d.	28
**2a**	C6:0‐solamine	C_18_H_39_N_3_O	3.51	314.3166 (0.1)	157.6623 (2.5)	66	17	4300
**2b**	C6:0‐solamine‐*N*‐oxide^b^	C_18_H_39_N_3_O_2_	3.79	330.3114 (−0.4)	165.6599 (2.8)	1	n.d.	73
**2c**	C6:0‐solamine‐di‐*N*‐oxide^b^	C_18_H_39_N_3_O_3_	4.02	346.3062 (−0.5)	173.6573 (2.4)	n.d	n.d.	16
**2d**	C6:0‐nor‐solamine^b^	C_17_H_37_N_3_O	3.42	300.3015 (1.8)	150.6545 (2.8)	n.d.	n.d.	50
**3**	C6:1‐solamine	C_18_H_37_N_3_O	3.19	312.3013 (1.1)	156.6545 (2.6)	4	2	51
**4**	C7:0‐solamine^b^	C_19_H_41_N_3_O	4.43	328.3320 (−0.8)	164.6700 (1.2)	9	9	150
**5**	C8:0‐solamine	C_20_H_43_N_3_O	5.33	342.3477 (−0.5)	171.6777 (0.5)	89	460	64
**6a**	C8:1‐solamine‐#1	C_20_H_41_N_3_O	4.70	340.3318 (−1.4)	170.6698 (0.4)	19	29	21
**6b**	C8:1‐solamine‐#2	C_20_H_41_N_3_O	5.01	340.3319 (−1.0)	170.6700 (1.2)	25	50	5
**7**	C8:2‐solamine	C_20_H_39_N_3_O	4.75	338.3166 (−0.1)	169.6622 (1.4)	49	14	6
**8**	C9:0‐solamine^b^	C_21_H_45_N_3_O	6.17	356.3637 (0.4)	178.6857 (1.6)	38	170	n.d.
**9**	C10:0‐solamine	C_22_H_47_N_3_O	6.94	370.3790 (−0.6)	185.6933 (0.1)	100	520	n.d.
**10a**	C10:1‐solamine	C_22_H_45_N_3_O	6.36	368.3634 (−0.4)	184.6855 (0.6)	1800	12 900	30
**10b**	C10:1‐solamine‐*N*‐oxide	C_22_H_45_N_3_O_2_	6.54	384.3580 (−1.1)	192.6831 (1.3)	55	200	n.d.
**10c**	C10:1‐solamine‐di‐*N*‐oxide	C_22_H_45_N_3_O_3_	6.65	400.3530 (−0.9)	200.6805 (0.6)	24	64	n.d.
**10d**	C10:1‐nor‐solamine^b^	C_21_H_43_N_3_O	6.29	354.3477 (−0.6)	177.6778 (1.1)	17	560	n.d.
**10e**	C10:1‐dinor‐solamine^b^	C_20_H_41_N_3_O	6.19	340.3325 (0.7)	170.6702 (2.5)	2	180	n.d.
**11**	C10:1‐O_1_‐solamine	C_22_H_45_N_3_O_2_	3.8–5.4^c^	384.3581 (−0.9)	192.6830 (0.9)	100	200	n.d.
**12a**	C10:2‐solamine‐#1	C_22_H_43_N_3_O	5.69	366.3478 (−0.3)	183.6776 (0.3)	490	460	n.d.
**12b**	C10:2‐solamine‐#2	C_22_H_43_N_3_O	6.33	366.3479 (0.1)	183.6779 (1.7)	290	7	64
**13**	C10:2‐O_1_‐solamine	C_22_H_43_N_3_O_2_	3.6–5.2^c^	382.3424 (−1.2)	191.6751 (0.1)	110	60	n.d.
**14**	C11:1‐solamine^b^	C_23_H_47_N_3_O	6.82	382.3793 (0.2)	191.6936 (1.7)	n.d.	n.d.	57
**15**	Benzoyl solamine^b^	C_19_H_33_N_3_O	1.89	320.2692 (−1.3)	160.6386 (0.7)	50	19	6
**16**	Dihydroferuloyl solamine^b^	C_22_H_39_N_3_O_3_	2.83	394.3060 (−1.5)	197.6567 (−0.6)	18	2	n.d.
**17a/b**	Cinnamoyl solamine	C_21_H_35_N_3_O	3.18/3.89^d^	346.2846 (−2.1)	173.6461 (−1.2)	1	7	3
**18a/b**	Coumaroyl solamine	C_21_H_35_N_3_O_2_	2.12/2.85^d^	362.2800 (−0.7)	181.6437 (−0.1)	19	1200	3
**18c/d**	Coumaroyl nor‐solamine^b^	C_20_H_33_N_3_O_2_	1.95/2.77^d^	348.2639 (−1.9)	174.6358 (−0.9)	n.d.	14	n.d.
**19a/b**	Caffeoyl solamine	C_21_H_35_N_3_O_3_	1.42/2.38^d^, ^e^	378.2748 (−0.9)	189.6414 (1.2)	7	3200	5
**19c/d**	Caffeoyl nor‐solamine^b^	C_20_H_33_N_3_O_3_	1.29/2.24^d^	364.2592 (−0.6)	182.6338 (2.2)	n.d.	75	n.d.
**20a/b**	Feruloyl solamine	C_22_H_37_N_3_O_3_	2.53/3.13^d^	392.2905 (−0.6)	196.6493 (1.3)	420	880	2
**20c/d**	Feruloyl nor‐solamine^b^	C_21_H_35_N_3_O_3_	2.45/3.06^d^, ^e^	378.2754 (0.7)	189.6414 (1.1)	n.d.	13	n.d.

Abbreviation: n.d., not detected.

^a^ The levels of individual acyl solamines were estimated using the calibration curve of synthetic C16:0‐solamine.

^b^ Compound previously not described in the literature.

^c^ Peak cluster containing up to 10 isomeric components with similar mass spectrometric behavior. *m/z* of [M + H]^+^ and [M + 2H]^2+^ were obtained from the most abundant isomer. For quantification, peaks of all isomers were integrated and summed up.

^d^ Pairs of *E/Z* isomers with similar mass spectrometric behavior, *m/z* of [M + H]^+^ and [M + 2H]^2+^ were obtained from the later eluting isomer. For quantification, peaks of both isomers were integrated and summed up.

^e^ **19b** co‐elutes with isomeric **20c**. Both compounds can be selectively detected in MS/MS mode using the transitions *m/z* 378.3 → *m/z* 163.039 (**19b**) and *m/z* 378.3 → *m/z* 177.055 (**20c**).

The detected MCFA solamines carry both even‐ and odd‐numbered fatty acyl moieties with chain lengths ranging from 6 to 11 and degrees of unsaturation varying from 0 to 2. The most abundant MCFA solamine in the tuber periderm of *S. tuberosum* and *S. pinnatisectum* is C10:1‐solamine (**10a**), whereas C6:0‐solamine (**2a**) is the most abundant one in the tuber periderm of *S. cardiophyllum* . Two chromatographically separable isomers were detected for C8:1‐solamine (**6a**/**b**) and for C10:2‐solamine (**12a**/**b**). In contrast, complex peak clusters with up to 10 isomeric components with highly similar CID tandem mass spectra were registered for the two MCFA solamines with oxygenated fatty acyl moieties: C10:1‐O_1_‐solamine (**11**) and C10:2‐O_1_‐solamine (**13**). In both cases, only the analytical data for the most abundant isomer are presented. Coumaroyl solamine (**18a/b**), caffeoyl solamine (**19a/b**) and feruloyl solamine (**20a/b**) were annotated as HCA solamines. High levels of these three compounds were particularly found in the tuber periderm of *S. pinnatisectum* . Minor acyl solamines with an aromatic acyl moiety include benzoyl solamine (**15**), dihydroferuloyl solamine (**16**), and cinnamoyl solamine (**17a/b**). Pairs of isomers with distinct retention times (Δt_R_ 0.7–1.0 min) and highly similar CID tandem mass spectra were detected for all four cinnamoyl solamines. Given the photochemical interconvertibility of geometric isomers of hydroxycinnamic acid conjugates, it seems probable that these isomer pairs represent *E*/*Z* isomers [16]. The reported analytical data for compounds **17a/b**–**20a/b** were obtained from the dominating, later eluting isomers.

All of the detected acyl solamine derivatives with a modified head group are related to the most abundant MCFA solamines **2a** and **10a** and to the HCA solamines **18a/b**, **19a/b**, and **20a/b**. Compared with their parent compounds, the concentration of these derivatives was generally low in the analyzed sample set. As with HCA solamines, pairs of isomers were also detectable for HCA nor‐solamines **18c/d**, **19c/d**, and **20c/d**. The only acyl dinor‐solamine that could be annotated was C10:1‐dinor‐solamine (**10e**). This was present at low concentrations in the tuber periderm of *S. tuberosum* and *S. pinnatisectum* . Acyl solamine derivatives with an oxygenated head group were found in the tuber periderm of *S. cardiophyllum* , including C6:0‐solamine‐*N*‐oxide (**2b**) and C6:0‐solamine‐di‐*N*‐oxide (**2c**), and in the tuber periderm of *S. tuberosum* and *S. pinnatisectum* , including C10:1‐solamine‐*N*‐oxide (**10b**) and C10:1‐solamine‐di‐*N*‐oxide (**10d**). Despite the high levels of HCA solamines in the tuber periderm of *S. pinnatisectum* , HCA solamine‐*N*‐oxides and HCA‐solamine‐di‐*N*‐oxides were not detected.

### 3.4 Retention Behavior of Fatty Acyl Solamines and Related Derivatives

The observed retention times of fatty acyl solamines generally follow the elution patterns of lipids in reversed‐phase chromatography [17]. The retention times of the annotated saturated fatty acyl solamines (C5:0‐C10:0) increased linearly with increasing chain length (Δt_R_/Δn_CH2_ ≈ 0.88 min). An increase in the number of double bond equivalents, as well as the oxygenation of the fatty acyl moiety, resulted in decreased retention times. In contrast, the gradual oxygenation of the two tertiary amino groups of the solamine head group (see, e.g., the compound series **10a**, **10b**, and **10c**) led to a slight and consistent increase in retention under the applied chromatographic conditions. Conversely, a gradual decrease in the number of *N*‐methyl groups in the head group (see compound series **10a**, **10d**, and **10e**) had the opposite effect on retention.

### 3.5 Semiquantification of Acyl Solamines and Related Derivatives in Tuber Periderm

Assuming that acyl solamines exhibit comparable molar response factors in ESI‐MS, an external calibration curve was established for the model compound C16:0‐solamine. This calibration curve was then used to semiquantify the levels of acyl solamines accumulating in the tuber periderm. The results of this estimation are shown in Table 1.

The limit of detection and the limit of quantification for C16:0‐solamine were determined to be 5 fmol and 20 fmol, respectively. This indicates that acyl solamines can be sensitively detected by UHPLC/ESI‐QTOFMS in positive ion mode. However, the dynamic working range was limited to two orders of magnitude (20–2000 fmol). Due to the wide concentration range of individual acyl solamines in the analyzed tuber periderm samples (three to four orders of magnitude), corresponding extracts had to be analyzed at different dilution levels. It should be noted that significant amounts of the surrogate calibrant (approximately 500 fmol) were absorbed by the chromatographic system when calibration solutions of C16:0‐solamine dihydrochloride in neat or acidified (0.5% formic acid) methanol were used, which renders calibration impossible. The use of matrix‐matched calibration solutions can overcome this problem. In this case, the absorption sites of the chromatographic system are occupied by other acyl solamines or chemically similar components from the matrix.

The total amount of acyl solamines in dry tuber periderm was estimated to be 1.4 mg/g in *S. tuberosum* ‘Quarta’, 7.4 mg/g in *S. pinnatisectum* WKS 31607, and 1.5 mg/g in *S. cardiophyllum* WKS 30086. The most abundant acyl solamine in the tuber periderm of *S. tuberosum* (**10a**), *S. pinnatisectum* (**10a**), and *S. cardiophyllum* (**2a**) accounted for 49%, 64%, and 89% of the total acyl solamine content, respectively. Although larger errors are to be expected, the calibration curve of C16:0‐solamine was used to estimate the amounts of acyl solamine derivatives with modified head group. These derivatives accounted for only 2.6% to 5.4% of the total acyl solamines content in dry tuber periderm, amounting to 0.04 mg/g in *S. tuberosum* ‘Quarta’, 0.4 mg/g in *S. pinnatisectum* WKS 31607, and 0.04 mg/g in *S. cardiophyllum* WKS 30086.

### 3.6 Fragmentation Behavior of Protonated Acyl Solamines

**FIGURE 3:**
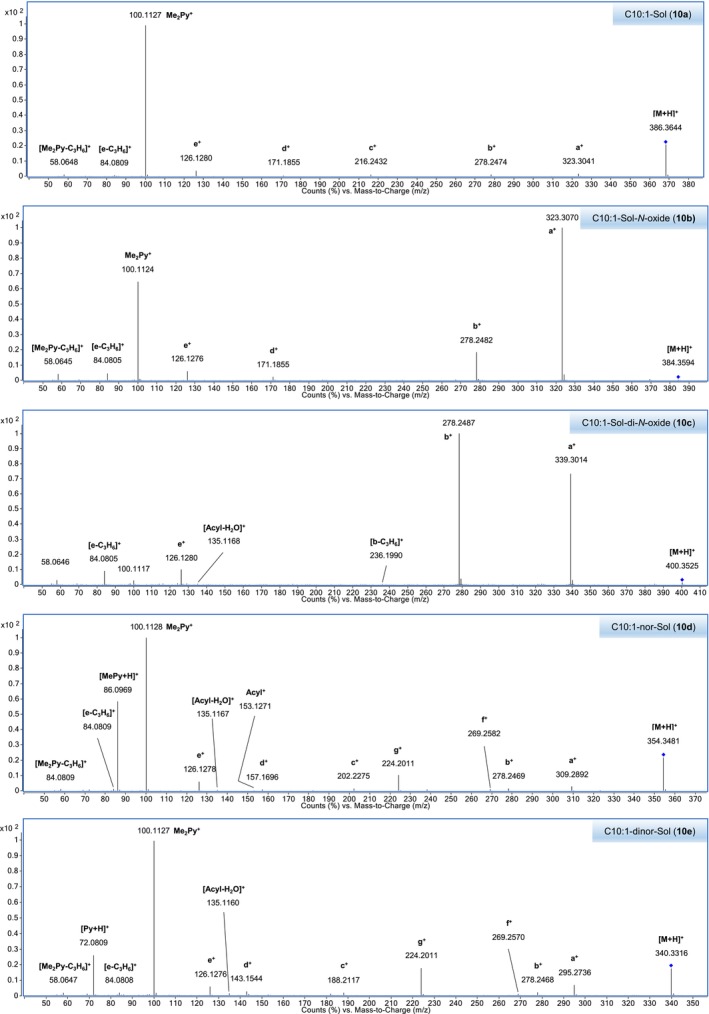
CID tandem mass spectra of C10:1‐solamine (**10a**) and corresponding derivatives with modified solamine head group (**10b‐e**). Spectra were obtained from [M + H]^+^ at a collision energy of 25 V using UHPLC/ESI‐QTOFMS and a tuber periderm extract of *S. pinnatisectum* WKS 31607. Precursor ions are marked with a blue diamond.

CID mass spectra of the singly protonated acyl solamines were acquired at CEs of 10, 25, and 40 V (Table S2). The CID mass spectra of the detected isomers of oxygenated MCFA solamines (**11** and **13**) and HCA solamines (**17a/b**–**20a/b**) were found to be very similar. Therefore, only the spectra of the most abundant isomer are reported. The 25 V‐CID mass spectrum of C10:1‐solamine (**10a**) is displayed in Figure 3.

**FIGURE 4:**
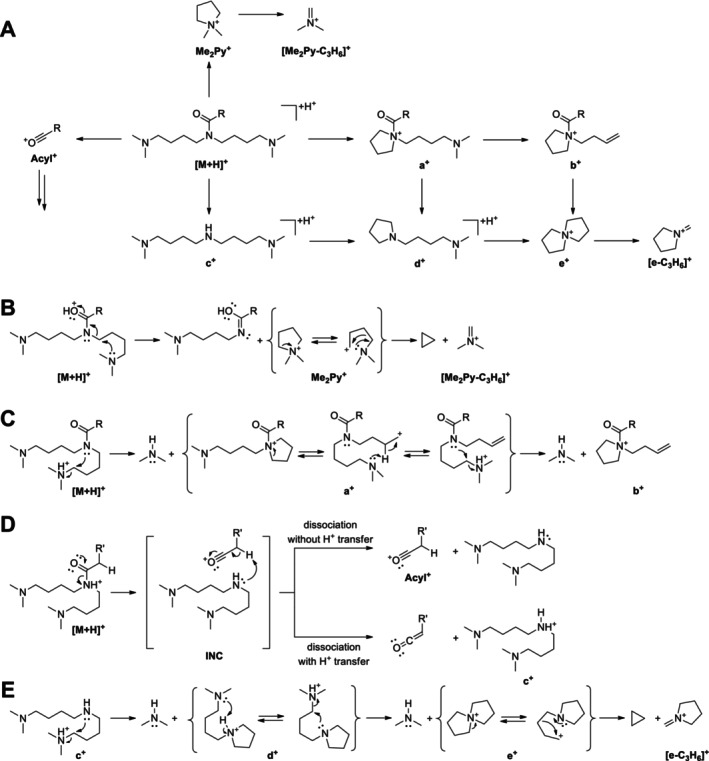
(A) Simplified fragmentation scheme of protonated acyl solamines under CID conditions. (B–E) Mechanistic proposals for selected fragmentation reaction sequences. INC, ion‐neutral complex.

Informative spectra were obtained in all cases at a CE of 25 V. With the exception of C10:2‐O_1_‐solamine (**13**), the investigated acyl solamines show a rather uniform fragmentation behavior (Table 2). To gain a deeper understanding of the main fragmentation pathways, pseudo‐MS^3^ spectra of the principal fragment ions of protonated **10a** (generated by in‐source fragmentation) were exemplarily acquired (Figure S1). The dominant fragmentation reaction of protonated acyl solamines is the formation of the characteristic *N,N*‐dimethylpyrrolidinium ion Me_2_Py^+^ at *m/z* 100.112, which appears at collision energies of 25 and 40 V as the base peak in all spectra (Figure 4). As indicated by pseudo‐MS^3^ analyses, Me_2_Py^+^ is also formed from the fragment ions a^+^, c^+^, and d^+^. Alternative fragmentation pathways of [M + H]^+^ include subsequent neutral losses of dimethylamine giving rise to a‐type ([M + H‐C_2_H_7_N]^+^) and b‐type ions ([M + H‐2C_2_H_7_N]^+^), which generally appear with relative intensities below 3% in nearly all spectra. The formation of complementary dimethylammonium ions at *m/z* 46.065 was not observed. The neutral loss of the acyl moiety from [M + H]^+^ results in the formation of c‐type ions [M + H^+^‐(RCO^+^‐H^+^)] at *m/z* 216.243, which consecutively eliminate two molecules of dimethylamine to give d‐type ions ([c‐C_2_H_7_N]^+^) at *m/z* 171.186 and e‐type ions ([c‐2C_2_H_7_N]^+^) at *m/z* 126.128. Alternatively, d‐ and e‐type ions can be formed from a‐ and b‐type ions by elimination of the acyl moiety. The ions c^+^, d^+^, and e^+^ appear in almost all spectra with low relative intensities (≤ 6%) similar to those observed for a^+^ and b^+^. At a CE of 40 V, significant ions at *m/z* 84.081 and *m/z* 58.065 were detected. As revealed by pseudo‐MS^3^ analyses, these ions are formed from e^+^ and Me_2_Py^+^ by elimination of C_3_H_6_, respectively. It should be noted that the electron ionization mass spectra of solapalmitine and solapalmitenine also display abundant ions at *m/z* 100, *m/z* 84, and *m/z* 58 [2]. At a CE of 25 V, CID mass spectra of protonated fatty acyl solamines exhibit, in some cases, low abundant (relative intensity ≤ 2%) acylium ions (RCO^+^) and related fragment ion species such as R^+^. In contrast, 25 V‐CID mass spectra of protonated C8:2‐solamine (**7**), benzoyl solamine (**15**), and cinnamoyl solamines (**17a/b**–**20a/b**) display acylium ions with significantly higher relative intensities (9%–70%). At a CE of 40 V, these resonance‐stabilized acylium ions undergo further fragmentation, yielding informative ions that reveal structural features of the acyl moiety. For instance, the 40 V‐CID mass spectrum of protonated **19b** shows fragment ions at *m/z* 163.039 (RCO^+^), *m/z* 145.028 ([RCO‐H_2_O]^+^), *m/z* 135.044 ([RCO‐CO]^+^), and *m/z* 117.033 ([RCO‐H_2_O‐CO]^+^), which are characteristic of a caffeoyl moiety (Table S2). In the case of dihydroferuloyl solamine (**16**), the formation of an acylium ion at *m/z* 179.070 was not observed. Instead, a related fragment ion of the type [RCO‐C_2_H_2_O]^+^ was detected at *m/z* 137.060.

**TABLE 2:** CID tandem mass spectral data of acyl solamines.

No.	*m/z* (relative intensity)								
	[M + H]^+^	a^+^	b^+^	c^+^	d^+^	e^+^	Me_2_Py^+^	Acyl^+^	Other ions
**1**	300 (6)	255 (1)	210 (2)	216 (1)	171 (0.2)	126 (3)	100 (100)	85 (0.3)	84 (2), 58 (1), 57 (0.4)
**2**	314 (8)	269 (1)	224 (2)	216 (0.3)	171 (0.3)	126 (3)	100 (100)	99 (0.1)	84 (1), 71 (0.2), 58 (1)
**3**	312 (5)	267 (1)	222 (2)	—	—	126 (2)	100 (100)	97 (2)	84 (1), 69 (0.1), 58 (1)
**4**	328 (10)	283 (1)	238 (1)	216 (1)	171 (0.3)	126 (3)	100 (100)	113 (0.1)	85 (0.2), 84 (1), 58 (1), 55 (0.8)
**5**	342 (14)	297 (2)	252 (1)	216 (1)	171 (0.3)	126 (3)	100 (100)	—	84 (1), 58 (1)
**6a**	340 (10)	295 (1)	250 (1)	216 (1)	171 (0.3)	126 (4)	100 (100)	—	84 (1), 58 (1), 55 (0.5)
**6b**	340 (9)	295 (1)	250 (1)	—	171 (0.1)	126 (2)	100 (100)	125 (1)	97 (0.2), 84 (0.3), 58 (1), 55 (0.5)
**7**	338 (6)	293 (1)	248 (1)	216 (1)	171 (0.3)	126 (3)	100 (100)	123 (9)	95 (4), 84 (0.3), 81 (2), 58 (1), 53 (0.5)
**8**	356 (18)	311 (2)	266 (1)	216 (1)	171 (1)	126 (3)	100 (100)	—	84 (1), 71 (0.2), 58 (1), 57 (0.3)
**9**	370 (28)	325 (2)	280 (1)	216 (1)	171 (0.5)	126 (4)	100 (100)	—	84 (1), 71 (0.2), 58 (1), 57 (0.3)
**10a**	368 (21)	323 (1)	278 (1)	216 (1)	171 (0.3)	126 (3)	100 (100)	—	84 (1), 69 (0.2), 58 (1), 55 (0.1)
**11** ^a^	384 (38)	339 (2)	—	216 (2)	171 (0.5)	126 (3)	100 (100)	—	84 (1), 58 (1)
**12a**	366 (17)	321 (1)	276 (1)	216 (1)	171 (0.5)	126 (3)	100 (100)	—	84 (1), 58 (1)
**12b**	366 (16)	321 (1)	276 (1)	216 (1)	171 (0.5)	126 (3)	100 (100)	151 (2)	133 (0.3), 84 (1), 81 (2), 69 (1), 67 (0.5), 58 (1)
**13** ^a^	382 (1)	—	—	216 (4)	171 (1)	126 (5)	100 (100)	—	364 (18), 319 (18), 274 (0.3), 84 (0.4), 58 (1)
**14**	382 (25)	337 (1)	292 (1)	216 (2)	171 (1)	126 (4)	100 (100)	—	84 (1), 58 (1)
**15**	320 (3)	275 (1)	230 (1)	—	—	126 (1)	100 (100)	105 (4)	84 (0.4), 77 (0.2), 58 (1)
**16**	394 (30)	349 (2)	304 (1)	216 (2)	171 (1)	126 (4)	100 (100)	—	137 (1), 84 (0.4), 58 (1)
**17a/b** ^b^	346 (8)	301 (1)	256 (0.5)	216 (1)	171 (1)	126 (5)	100 (100)	131 (13)	103 (2), 84 (0.5), 58 (1)
**18a/b** ^b^	362 (12)	317 (2)	272 (0.3)	216 (3)	171 (1)	126 (5)	100 (100)	147 (39)	119 (2), 91 (1), 84 (1), 58 (1)
**19a/b** ^b^	378 (17)	333 (1)	288 (0.3)	216 (3)	171 (1)	126 (6)	100 (100)	163 (28)	145 (1), 135 (1), 117 (0.4), 84 (0.2), 58 (0.5)
**20a/b** ^b^	392 (20)	347 (1)	302 (0.2)	216 (6)	171 (2)	126 (5)	100 (100)	177 (70)	149 (0.4), 145 (7), 117 (1), 84 (0.1), 58 (1)

*Note:* Mass spectra were obtained from [M + H]^+^ at a collision energy of 25 V using UHPLC/ESI‐QTOFMS. More detailed information can be found in Table S2.

^a^ Up to 10 isomeric components with similar CID mass spectra. Reported mass spectral data were obtained from the most abundant isomer.

^b^ Pairs of *E/Z* isomers with similar CID mass spectra. Reported mass spectral data were obtained from the later eluting isomer.

As mentioned above, the fragmentation behavior of C10:2‐O_1_‐solamine (**13**) slightly deviates from that of the other acyl solamines (Table 2). The initial fragmentation reaction of protonated **13** is the elimination of water from the oxygenated, unsaturated fatty acyl side chain. The [M + H‐H_2_O]^+^ ions formed subsequently eliminate two molecules of dimethylamine, resulting in a‐ and b‐type ions, which are accordingly both shifted by −18.011 u in relation to [M + H]^+^. Other fragment ions without fatty acyl moiety (c^+^, d^+^, e^+^, and Me_2_Py^+^) appear at the same *m/z* as observed for the other acyl solamines.

It should be noted that doubly protonated acyl solamines can also be used as precursor ions in CID experiments. Due to the presence of two charges, more complex product ion spectra are obtained in this case. At a CE of 12.5 V, the CID mass spectrum of doubly protonated **10a** displays Me_2_Py^+^ as the base peak (Figure S2). Consecutive elimination of Me_2_Py^+^ and dimethylamine from [M + 2H]^2+^ results in singly charged f‐type [M + 2H‐Me_2_Py]^+^ and g‐type ions [f‐C_2_H_7_N]^+^, which were not observed in the CID mass spectra of singly protonated **10a**. A‐type ([M + 2H‐C_2_H_7_N]^+^), c‐type ([M + 2H^+^‐(RCO^+^‐H^+^)]) and d‐type ions ([c‐C_2_H_7_N]^2+^) appear as doubly charged, e‐type ions ([d^2+^‐C_2_H_8_N^+^]) as singly charged product ions. In contrast to singly protonated **10a**, CID of doubly protonated **10a** yields dimethylammonium ions at *m/z* 46.065 as product ions.

### 3.7 Fragmentation Behavior of Protonated Acyl Solamine‐*N*‐oxides and Di‐*N*‐oxides

**FIGURE 5:**
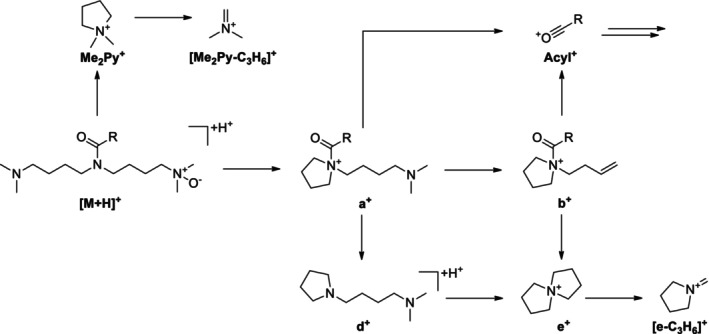
Simplified fragmentation scheme of protonated acyl solamine‐*N*‐oxides under CID conditions.

**FIGURE 6:**
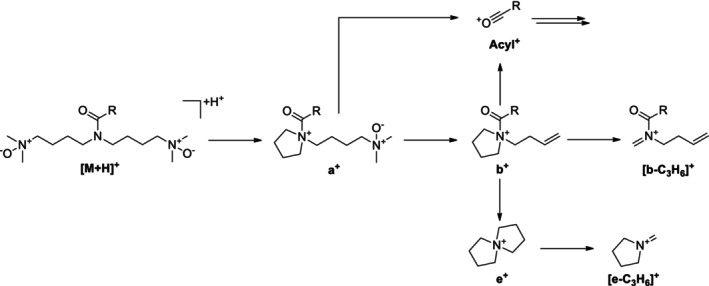
Simplified fragmentation scheme of protonated acyl solamine‐di‐*N*‐oxides under CID conditions.

CID mass spectra of the singly protonated acyl solamine‐*N*‐oxides (**2b** and **10b**) and acyl solamine‐di‐*N*‐oxides (**2c** and **10c**) were acquired at CEs of 10, 25, and 40 V (Table 3 and Tables S3 and S4). Figure 3 shows the 25 V‐CID mass spectra of C10:1‐solamine‐*N*‐oxide (**10b**) and C10:1‐solamine di‐*N*‐oxide (**10c**); Figures 5 and 6 depict simplified fragmentation schemes.

**TABLE 3:** CID tandem mass spectral data of acyl solamine‐*N*‐oxides and acyl solamine‐di‐*N*‐oxides.

No.	*m/z* (relative intensity)										
	[M + H]^+^	a^+^	b^+^	[b‐C_3_H_6_]^+^	d^+^	e^+^	Me_2_Py^+^	[e‐C_3_H_6_]^+^	[Me_2_Py‐C_3_H_6_]^+^	Acyl^+^	Other ions
**2b**	330 (0.1)	269 (91)	224 (39)	—	171 (4)	126 (9)	100 (100)	84 (11)	58 (8)	99 (1)	—
**2c**	346 (0.3)	285 (37)	224 (100)	182 (1)	—	126 (9)	100 (2)	84 (13)	58 (3)	99 (1)	71 (0.4)
**10b**	384 (0.3)	323 (100)	278 (19)	—	171 (2)	126 (6)	100 (64)	84 (4)	58 (4)	—	—
**10c**	400 (1.2)	339 (74)	278 (100)	236 (0.2)	—	126 (9)	100 (3)	84 (9)	58 (8)	—	135 (0.5)

*Note:* Mass spectra were obtained from [M + H]^+^ at a collision energy of 25 V using UHPLC/ESI‐QTOFMS. More detailed information can be found in Tables S3 and S4.

At CEs of 25 V and 40 V, CID mass spectra of protonated **2b** and **10b** display abundant Me_2_Py^+^ ions. In contrast to protonated acyl solamines, CID of protonated **2b** and **10b** leads to the formation of highly abundant a‐type ions ([M + H‐C_2_H_7_NO]^+^) at CEs of 10 and 25 V, and also to moderately abundant b‐type ions ([M + H‐C_2_H_7_NO‐C_2_H_7_N]^+^) at a CE of 25 V. The alternative a‐type ion ([M + H‐C_2_H_7_N]^+^) is not formed. D‐type and e‐type ions are registered at *m/z* 171.186 and *m/z* 126.128. The formation of c‐type ions at *m/z* 232.238 is not observed. At a CE of 40 V, the ions [e‐C_3_H_6_]^+^ at *m/z* 84.081 and [Me_2_Py‐C_3_H_6_]^+^ at *m/z* 58.065 increase in relative intensity, and acylium ions and derived fragment ions are registered.

In the CID mass spectra of the protonated **2c** and **10c**, the characteristic ion Me_2_Py^+^ at *m/z* 100.112 is virtually absent. The formation of analogous oxygenated fragment ions at *m/z* 116.107 is not observed. CID mass spectra of protonated **2c** and **10c** display intense a‐type ions ([M + H‐C_2_H_7_NO]^+^) at CEs of 10 V and 25 V, intense b‐type ions ([M + H‐2C_2_H_7_NO]^+^) and moderately intense e‐type ions at CEs of 25 V and 40 V. C‐type and d‐type ions are not formed. At a CE of 40 V, the formation of acylium ions and derived fragment ions from b‐type ions is observed. Further fragmentation of b‐ and e‐type ions leads to the formation of low abundant [b‐C_3_H_6_]^+^ and high abundant [e‐C_3_H_6_]^+^ ions. The latter appear as base peak in the 40 V‐CID mass spectra of protonated **2c** and **10c**.

### 3.8 Fragmentation Behavior of Protonated Acyl Nor‐solamines and Dinor‐solamines

**FIGURE 7:**
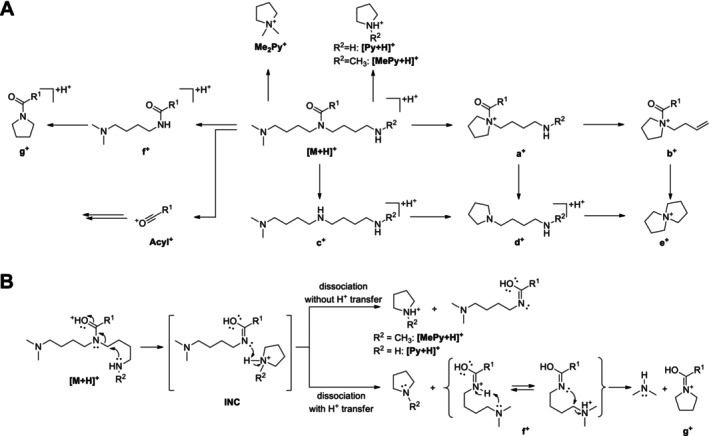
(A) Simplified fragmentation scheme of protonated acyl nor‐solamines (R^2^ = CH_3_) and protonated acyl dinor‐solamines (R^2^ = H) under CID conditions. (B) Mechanistic proposal for the formation the fragment ions [MePy+H]^+^, [Py + H]^+^, f^+^ and g^+^ starting from [M + H]^+^. INC, ion‐neutral complex.

CID mass spectra of the singly protonated acyl nor‐solamines (**2d**, **10d**, **18c/d**, **19c/d**, and **20c/d**) and the acyl dinor‐solamine **10e** were acquired at CEs of 10, 25, and 40 V (Table 4, Tables S5 and S6). CID mass spectra of the isomeric HCA nor‐solamines (**18c/d**–**20c/d**) are highly similar and are therefore only given for the later eluting isomers. Figure 3 shows the 25 V‐CID mass spectra of C10:1‐nor‐solamine (**10d**) and C10:1‐dinor‐solamine (**10e**); Figure 7 shows a simplified fragmentation scheme.

**TABLE 4:** CID tandem mass spectral data of acyl nor‐solamines and acyl dinor‐solamine **10e**.

No.	*m/z* (relative intensity)											
	[M + H]^+^	a^+^	b^+^	f^+^	g^+^	c^+^	d^+^	e^+^	Me_2_Py^+^	[MePy+H]^+^	[Py+H] ^+^	Acyl^+^
**2d**	300 (8)	255 (2)	224 (3)	215 (1)	170 (10)	202 (1)	157 (1)	126 (7)	100 (100)	86 (75)	—	99 (1)
**10d**	354 (21)	309 (3)	278 (1)	269 (1)	224 (10)	202 (1)	157 (1)	126 (6)	100 (100)	86 (59)	—	153 (0.1)
**18c/d** ^a^	348 (9)	303 (5)	272 (1)	263 (1)	218 (8)	202 (5)	157 (3)	126 (13)	100 (100)	86 (43)	—	147 (90)
**19c/d** ^a^	364 (12)	319 (4)	288 (1)	279 (1)	234 (7)	202 (4)	157 (3)	126 (11)	100 (100)	86 (41)	—	163 (61)
**20c/d** ^a^	378 (13)	333 (4)	—	293 (1)	248 (4)	202 (7)	157 (3)	126 (7)	100 (79)	86 (23)	—	177 (100)
**10e**	340 (17)	295 (6)	278 (2)	269 (1)	224 (17)	188 (1)	143 (2)	126 (8)	100 (100)	—	72 (39)	—

*Note:* Mass spectra were obtained from [M + H]^+^ at a collision energy of 25 V using UHPLC/ESI‐QTOFMS. More detailed information can be found in Tables S5 and S6.

^a^ Pairs of *E/Z* isomers with similar CID mass spectra. Reported mass spectral data were obtained from the later eluting isomer.

At CEs of 25 and 40 V, CID mass spectra of acyl nor‐solamines display prominent Me_2_Py^+^ ions at *m/z* 100.112 and homologous *N*‐methylpyrrolidinium ions ([MePy+H^+^]) at *m/z* 86.096. Low abundant a‐type ([M + H‐C_2_H_7_N]^+^) and b‐type ions ([M + H‐C_2_H_7_N‐CH_5_N]^+^) are formed at a CE of 25 V. Formation of the alternative a‐type ions ([M + H‐CH_5_N]^+^) is not observed. Compared with protonated acyl solamines, c‐type and d‐type ions formed from protonated acyl nor‐solamines are shifted by −14.016 u and detected at a CE of 25 V as low abundant peaks at *m/z* 202.228 and *m/z* 157.170, respectively. In contrast, e‐type ions appeared without shift at *m/z* 126.128. The 25 V‐CID mass spectra of protonated acyl nor‐solamines exhibit low abundant f‐type ([M + H‐MePy]^+^) and g‐type ([f‐C_2_H_7_N]^+^) ions, which are formed by subsequent neutral losses of methylpyrrolidine and dimethylamine from [M + H]^+^. Both f‐type and g‐type ions are absent in the CID mass spectra of singly protonated acyl solamines, solamine‐*N*‐oxides, and solamine‐di‐*N*‐oxides. At a CE of 40 V, the above‐mentioned fragmentation products of acylium, e‐type and Me_2_Py^+^ ions appear with increased relative intensities.

The fragmentation pattern of the protonated fatty acyl dinor‐solamine **10e** is very similar to that of the corresponding protonated fatty acyl nor‐solamine **10d**. Due to the lack of an *N*‐methyl group, several fragment ions are characteristically shifted by the mass of CH_2_.The 25 V‐ and 40 V‐CID mass spectra of **10e** are dominated by Me_2_Py^+^ ions at *m/z* 100.112 and pyrrolidinium ions ([Py + H^+^]) at *m/z* 72.081. This clearly indicates that the dinor‐solamine moiety of **10e** carries one dimethylamino and one amino moiety rather than two methylamino moieties. Compared with **10d**, a‐type ([M + H‐C_2_H_7_N]^+^), c‐type ([M + H^+^‐(RCO^+^‐H^+^)]) and d‐type ([c‐C_2_H_7_N]^+^) ions formed from protonated **10e** were found to be shifted by −14.016 u. In contrast, b‐type, e‐type, f‐type, and g‐type ions appeared at the same *m/z* in both spectra.

### 3.9 Structural Elucidation of Unsaturated MCFA Solamines From Potato Tuber Periderm

With estimated dry matter concentrations of 0.66, 0.18, and 0.11 mg/g, C10:1‐solamine (**10a**) and the two isomeric C10:2‐solamines **12a** and **12b** were the three most abundant fatty acyl solamines in potato tuber periderm of the cultivar ‘Quarta’. Due to the importance of potato as a major food crop, further structural elucidation of these three metabolites is desirable. To determine the C‐C double bond positions in unsaturated lipids using CID tandem mass spectrometry, epoxidation with meta‐chloroperbenzoic acid (m‐CPBA) was proposed as a derivatization method [18, 19]. As previously shown, this strategy can also be applied for the localization of C‐C double bonds in LCFA solamines (e.g., C16:2(7,10)‐solamine) [9]. In addition to the epoxidation of the unsaturated fatty acyl moiety, the two terminal dimethylamino moieties are converted into their respective *N*‐oxides.

To localize the C‐C double bonds in the fatty acyl moieties of **10a**, **12a**, and **12b**, small quantities of these MCFA solamines were isolated from potato tuber periderm using a combination of strong cation exchange solid‐phase extraction and reversed‐phase preparative HPLC. Whereas **12a** was obtained in almost pure form, semipreparative HPLC failed to separate the nearly co‐eluting **10a** and **12b**.

**FIGURE 8:**
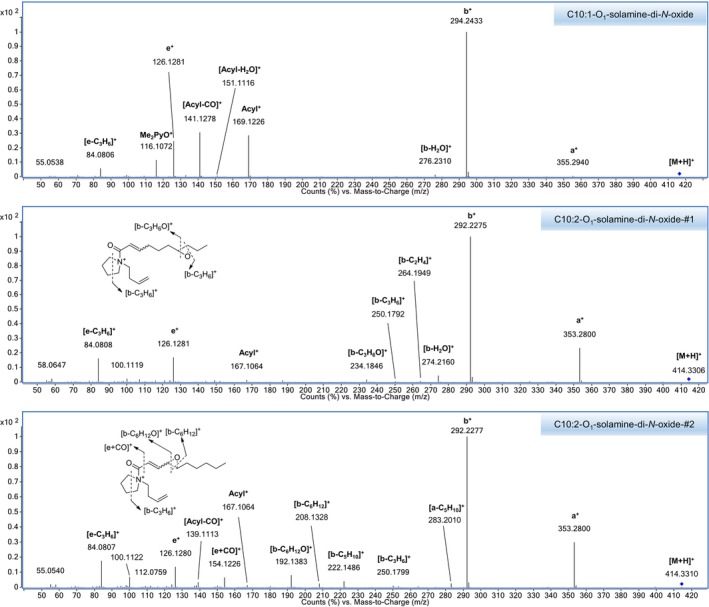
CID tandem mass spectra of the oxidation products obtained from reaction of C10:1‐solamine (**10a**, upper panel), C10:2‐solamine‐#1 (**12a**, middle panel), and C10:2‐solamine‐#2 (**12b**, lower panel) with meta‐chloroperbenzoic acid. Spectra were recorded in positive ion mode using UHPLC/ESI‐QTOFMS at a collision energy of 30 V. Precursor ions ([M + H^+^]) are marked with a blue diamond.

The isolated mixture of **10a** and **12b** was reacted with m‐CPBA and analyzed by UHPLC/ESI‐QTOFMS in positive ion mode. Besides other reaction products, protonated triply oxygenated products **10a‐O**_**3**_ (t_R_ = 3.02 min) and **12b‐O**_**3**_ (t_R_ = 5.52 min) were detected at *m/z* 416.3474 (mass error: 2.1 ppm) and *m/z* 414.3329 (mass error: −0.6 ppm). The 20 V‐CID mass spectra of protonated **10a‐O**_**3**_ and **12b‐O**_**3**_ display intense a‐type and b‐type ions of the form [M + H‐C_2_H_7_NO]^+^ and [M + H‐2C_2_H_7_NO]^+^, suggesting that both products are singly oxygenated fatty acyl solamine‐di‐*N*‐oxides. The 30 V‐CID mass spectrum of protonated **10a‐O**_**3**_ shows abundant acylium ions at *m/z* 169.123 (RCO^+^) and associated fragment ions at *m/z* 151.112 ([RCO‐H_2_O]^+^) and *m/z* 141.128 (R^+^) (Figure 8). The large retention time shift between **10a‐O**_**3**_ and **10a** and the absence of oxirane ring cleavage products in the CID mass spectrum of protonated **10a‐O**_**3**_ suggest that **10a‐O**_**3**_ is a rearrangement product of a primarily formed reactive oxirane derivative. Therefore, it can be hypothesized that the C‐C double bond of **10a** is conjugated to the amide carbonyl. To verify this hypothesis, C10:1(2*E*)‐solamine dihydrochloride and C10:1(2*Z*)‐solamine dihydrochloride were synthesized from solamine and commercially available (2*E*)‐decenoic acid and (2*Z*)‐decenoic acid. Synthetic C10:1(2*Z*)‐solamine and **10a** from extracts of *S. tuberosum* and *S. pinnatisectum* possess identical chromatographic and mass spectral properties suggesting that **10a** is C10:1(2*Z*)‐solamine (Figure S3). However, definitive proof of the structure of **10a** requires NMR spectroscopic investigations.

The 30 V‐CID mass spectrum of protonated **12b‐O**_**3**_ displays b‐type derived fragment ions at *m/z* 222.149 ([**b** ^+^ **‐**C_5_H_10_]), *m/z* 208.133 ([**b** ^+^ **‐**C_6_H_12_]), and *m/z* 192.138 ([**b** ^+^ **‐**C_6_H_12_O]) (Figure 8). The latter two are associated with a cleavage of an oxirane ring encompassing C‐4 and C‐5 of the fatty acyl moiety. The observed neutral losses associated with the oxirane ring cleavage furthermore suggest that the second C‐C double bond is located between C‐2 and C‐3. In order to obtain a UV spectrum of **12b**, analytical separation of the mixture of **10a** and **12b** was attempted. Usage of 0.3% formic acid in methanol instead of 0.5% formic acid in acetonitrile as organic modifier and modification of the gradient program resulted in the separation of **10a** and **12b** (Figure S4). The extracted UV spectrum of **12b** (Figure S4) shows an absorption maximum at 275 nm, similar to that described for (2*E*,4*Z*)‐decadienoic acid (λ_max_ = 265 nm) [20]. Based on this observation and the CID mass spectrum of protonated **12b‐O**_**3**_, it is suggested that **12b** is C10:2(2,4)‐solamine.

In addition to other products, the reaction of **12a** with m‐CPBA resulted in a triply oxidized product **12a‐O**_**3**_ (t_R_ = 4.42 min) which was detected in its protonated form at *m/z* 414.3325 (mass error: 0.3 ppm). At a CE of 20 V, the CID mass spectrum of protonated **12a‐O**_**3**_ displays intense a‐type and b‐type ions of the form [M + H‐C_2_H_7_NO]^+^ and [M + H‐2C_2_H_7_NO]^+^ indicating that **12a‐O**_**3**_ is a singly oxygenated fatty acyl solamine‐di‐*N*‐oxide. The 30 V‐CID mass spectrum of protonated **12a‐O**_**3**_ displays b‐type derived fragment ions at *m/z* 264.195 ([**b‐**C_2_H_4_]^+^), *m/z* 250.179 ([**b**‐C_3_H_6_]^+^), and *m/z* 234.185 ([**b‐**C_3_H_6_O]^+^) (Figure 8). This suggests that **12a‐O**_**3**_ is epoxidized at C‐7 and C‐8 and that the second double bond is located somewhere between C‐1 and C‐7. Unfortunately, the reaction of **12a** with m‐CPBA did not yield other oxidation products, which allow direct deduction of the position of the second double bond.

## 4 Conclusion

Reversed‐phase UHPLC/ESI‐QTOFMS operated in positive ion mode enables rapid separation and sensitive detection of acyl solamines from plant extracts. Due to their characteristic ionization behavior (formation of singly and doubly protonated molecules) and uniform fragmentation behavior (formation of Me_2_Py^+^ at *m/z* 100.112), data‐independent data acquisition methods, such as all‐ion fragmentation, can be readily applied to screen for these alkaloids. It can be anticipated that data‐dependent data acquisition approaches (auto‐MS/MS) will also be suitable for screening. Acyl solamine derivatives with *N*‐oxidized or *N*‐demethylated solamine head groups show a very similar ionization and fragmentation behavior and can be detected by the same method via the fragment ion e^+^ at *m/z* 126.128 or, with the exception of acyl solamine‐di‐*N*‐oxides, also via the fragment ion Me_2_Py^+^. CID mass spectra of protonated acyl solamines and related derivatives enable the structural characterization of the solamine head group. Therefore, the presence of the ions Me_2_Py^+^, [MePy+H]^+^, and [Py + H]^+^ as well as neutral losses between [M + H]^+^, a^+^, and b^+^ can be evaluated. In contrast to protonated fatty acyl solamines, protonated hydroxycinnamoyl solamines form abundant acyl ions and derived fragment ions upon CID, which enables the structural characterization of the acyl moiety. For a more detailed structural characterization of unsaturated fatty acyl solamines, oxidation with m‐CPBA is required prior to tandem mass spectrometric analysis. However, further studies are needed to elucidate the reaction behavior of α,β‐unsaturated fatty acyl solamines towards m‐CPBA. Overall, the developed methods and the compiled analytical data represent valuable resources for further investigation of the occurrence, structural diversity, and biological activity of acyl solamines and related derivatives in plants of the genus *Solanum*.

## Conflicts of Interest

The authors declare no conflicts of interest.

## Supporting information


**Supporting Information S1:** NMR data of synthetic fatty acyl solamine dihydrochlorides.
**Figure S1:** CID tandem mass spectra (MS^2^ and pseudo‐MS^3^) of protonated **10a**.
**Figure S2:** CID tandem mass spectrum of doubly protonated **10a**.
**Figure S3:** Authentication of **10a** as C10:1(2*Z*)‐solamine.
**Figure S4:** Chromatographic separation of **10a** and **12b**, UV spectrum of **12b**.
**Table S1:** Chromatographic peak characteristics of C16:0‐solamine.
**Table S2:** Analytical data of acyl solamines.
**Table S3:** Analytical data of acyl solamines‐*N*‐oxides.
**Table S4:** Analytical data of acyl solamines‐di‐*N*‐oxides.
**Table S5:** Analytical data of acyl nor‐solamines.
**Table S6:** Analytical data of acyl dinor‐solamine **10e**.

## Data Availability

The data that support the findings of this study are available in the Supporting Information of this article.
