# Supplementary material for: Analysis of Acyl Solamines in Tuber Periderm of Cultivated and Wild Potatoes Using Liquid Chromatography Coupled With Electrospray Ionization Quadrupole Time‐of‐Flight Mass Spectrometry
Source: J Mass Spectrom. 2025 Sep 21;60(10):e5177. doi: 10.1002/jms.5177 (PMC12450587; doi:10.1002/jms.5177)
Supplement: Supplementary file 1 — Supporting Information S1: NMR data of synthetic fatty acyl solamine dihydrochlorides. Figure S1: CID tandem mass spectra (MS2 and pseudo‐MS3) of protonated 10a. Figure S2: CID tandem mass spectrum of doubly protonated 10a. Figure S3: Authentication of 10a as C10:1(2Z)‐solamine. Figure S4: Chromatographic separation of 10a and 12b, UV spectrum of 12b. Table S1: Chromatographic peak characteristics of C16:0‐solamine. Table S2: Analytical data of acyl solamines. Table S3: Analytical data of acyl solamines‐N‐oxides. Table S4: Analytical data of acyl solamines‐di‐N‐oxides. Table S5: Analytical data of acyl nor‐solamines. Table S6: Analytical data of acyl dinor‐solamine 10e. [file JMS-60-e5177-s001.pdf]

Supporting Information

Analysis of acyl solamines in tuber periderm of cultivated and wild potatoes using liquid chromatography coupled with electrospray ionization quadrupole time-of-flight mass spectrometry

Christoph Böttcher*, Karin Gorzolka, Paul Himmighofen, Torsten Meiners

Julius Kühn-Institute, Federal Research Centre for Cultivated Plants, Institute for Ecological Chemistry, Plant Analysis and Stored Product Protection, Königin-Luise-Strasse 19, 14195 Berlin, Germany

*Corresponding author. Tel.: +49 30 8304 2386, Fax: +49 30 8304 2503
E-mail addresses: Christoph.Boettcher@julius-kuehn.de, Karin.Gorzolka@julius-kuehn.de, Paul.Himmighofen@julius-kuehn.de, Torsten.Meiners@julius-kuehn.de

NMR spectroscopic characterization of synthesized fatty acyl solamine dihydrochlorides

NMR spectra were recorded using a JEOL ECZ-600 NMR spectrometer equipped with a 5 mm liquid nitrogen cryo probe or a Bruker AVANCE III 700 NMR spectrometer equipped with a 5 mm helium cryo probe. All spectra were referenced to the solvent signal of CD_3OD at δ_H 3.31 and δ_C 49.15, respectively. Chemical shifts (δ) are reported in ppm, coupling constants (*J*) in Hz.

Hexadecanoyl-solamine dihydrochloride (C16:0-solamine × 2 HCl).

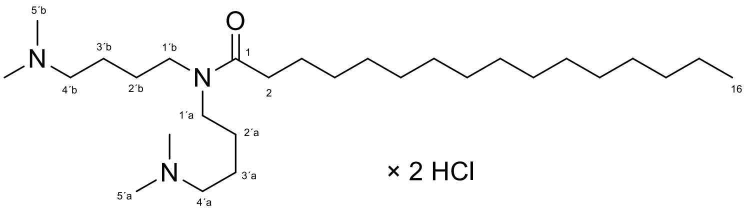

^{1}H NMR (600 MHz, CD_3OD, 298 K): δ 3.44-3.38 (m, 4H, 1´a-CH_2, 1´b-CH_2), 3.22-3.16 (m, 4H, 4´a-CH_2, 4´b-CH_2), 2.90 (s, 6H, 5´a-CH_3 or 5´b-CH_3), 2.89 (s, 6H, 5´a-CH_3 or 5´b-CH_3), 2.40 (t, *J* = 7.5 Hz, 2H, 2-CH_2), 1.81-1.57 (m, 10H, 2´a-CH_2, 2´b-CH_2, 3´a-CH_2, 3´b-CH_2, 3-CH_2), 1.40-1.23 (m, 24H, 4-CH_2, 5-CH_2, 6-CH_2, 7-CH_2, 8-CH_2, 9-CH_2, 10-CH_2, 11-CH_2, 12-CH_2, 13-CH_2, 14-CH_2, 15-CH_2), 0.90 (t, *J* = 7.0 Hz, 3H, 16-CH_3).

^{13}C NMR (151 MHz, CD_3OD, 298 K): δ 175.79 (1-CO), 58.72 (4´a-CH_2 or 4´b-CH_2), 58.66 (4´a-CH_2 or 4´b-CH_2), 48.81 (1´a-CH_2 or 1´b-CH_2), 46.24 (1´a-CH_2 or 1´b-CH_2), 43.59 (5´a-CH_3 or 5´b-CH_3), 43.56 (5´a-CH_3 or 5´b-CH_3), 34.11 (2-CH_2), 33.19 (14-CH_2), 30.9-30.6 (4-CH_2, 5-CH_2, 6-CH_2, 7-CH_2, 8-CH_2, 9-CH_2, 10-CH_2, 11-CH_2, 12-CH_2, 13-CH_2), 27.14 (2´a-CH_2 or 2´b-CH_2), 26.87 (3-CH_2), 25.77 (2´a-CH_2 or 2´b-CH_2), 23.85 (15-CH_2), 23.15 (3´a-CH_2 or 3´b-CH_2), 23.08 (3´a-CH_2 or 3´b-CH_2), 14.60 (16-CH_3).

(2*E*)-Dec-2-enoyl-solamine dihydrochloride (C10:1(2*E*)-solamine × 2 HCl).

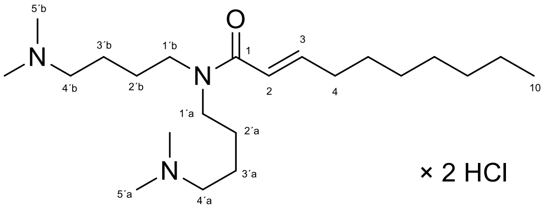

^{1}H NMR (700 MHz, CD_3OD, 298 K): δ 6.85 (dt, J = 14.5, 7.0 Hz, 1H, 3-CH), 6.42 (d, J = 14.9 Hz, 1H, 2-CH), 3.50 (br. t, J = 6.5 Hz, 2H, 1´a-CH_2 or 1´b-CH_2), 3.47 (br. t, J = 6.4 Hz, 2H, 1´a-CH_2 or 1´b-CH_2), 3.22 - 3.17 (m, 4H, 4´a-CH_2, 4´b-CH_2), 2.90 (s, 6H, 5´a-CH_3 or 5´b-CH_3), 2.89 (s, 6H, 5´a-CH_3 or 5´b-CH_3), 2.27 (q, J = 7.3 Hz, 2H, 4-CH_2), 1.83-1.63 (m, 8H, 2´a-CH_2, 2´b-CH_2, 3´a-CH_2, 3´b-CH_2), 1.48 (p, J = 7.1 Hz, 2H, 5-CH_2), 1.39-1.26 (m, 8H, 6-CH_2, 7-CH_2, 8-CH_2, 9-CH_2), 0.90 (t, J = 7.0 Hz, 3H, 10-CH_3).

^{13}C NMR (176 MHz, CD_3OD, 298 K): δ 169.06 (1-CO), 148.89 (3-CH), 121.58 (2-CH), 58.79 (4´a-CH_2 or 4´b-CH_2), 58.73 (4´a-CH_2 or 4´b-CH_2), 48.72 (1´a-CH_2 or 1´b-CH_2), 46.90 (1´a-CH_2 or 1´b-CH_2), 43.67 (5´a-CH_3 or 5´b-CH_3), 43.65 (5´a-CH_3 or 5´b-CH_3), 33.61 (4-CH_2), 33.09 (8-CH_2), 30.48 (6-CH_2 or 7-CH_2), 30.36 (6-CH_2 or 7-CH_2), 29.87 (5-CH_2), 27.65 (2´a-CH_2 or 2´b-CH_2), 25.89 (2´a-CH_2 or 2´b-CH_2), 23.81 (9-CH_2), 23.14 (3´a-CH_2, 3´b-CH_2), 14.55 (10-CH_3).

(2*Z*)-Dec-2-enoyl-solamine dihydrochloride (C10:1(2*Z*)-solamine × 2 HCl).

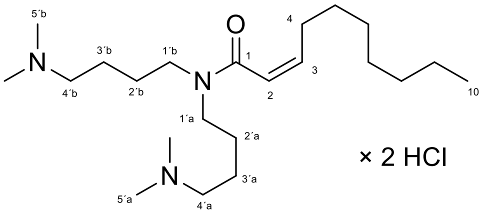

^1H NMR (700 MHz, CD_3OD, 298 K) δ 6.16 (dt, J = 11.6, 1.6 Hz, 1H, 2-CH), 6.00 (dt, J = 11.6, 7.4 Hz, 1H, 3-CH), 3.48-3.42 (m, 4H, 1´a-CH_2, 1´b-CH_2), 3.23-3.16 (m, 4H, 4´a-CH_2, 4´b-CH_2), 2.89 (s, 12H, 5´a-CH_3, 5´b-CH_3), 2.32 (q, J = 7.3 Hz, 2H, 4-CH_2), 1.79-1.64 (m, 8H, 2´a-CH_2, 2´b-CH_2, 3´a-CH_2, 3´b-CH_2), 1.44 (p, J = 7.2 Hz, 2H, 5-CH_2), 1.36-1.26 (m, 8H, 6-CH_2, 7-CH_2, 8-CH_2, 9-CH_2), 0.90 (t, J = 7.1 Hz, 3H, 10-CH_3).

^{13}C NMR (176 MHz, CD_3OD, 298 K) δ 170.29 (1-CO), 143.62 (3-CH), 122.98 (2-CH), 58.72 (4´a-CH_2 or 4´b-CH_2), 58.66 (4´a-CH_2 or 4´b-CH_2), 49.33 (below solvent signal, 1´a-CH_2 or 1´b-CH_2), 45.84 (1´a-CH_2 or 1´b-CH_2), 43.61 (5´a-CH_2, 5´b-CH_2), 33.09 (8-CH_2), 30.65 (4-CH_2 or 5-CH_2 or 6-CH_2 or 7-CH_2), 30.59 (4-CH_2 or 5-CH_2 or 6-CH_2 or 7-CH_2), 30.37 (4-CH_2 or 5-CH_2 or 6-CH_2 or 7-CH_2), 30.30 (4-CH_2 or 5-CH_2 or 6-CH_2 or 7-CH_2), 27.07 (2´a-CH_2 or 2´b-CH_2), 25.85 (2´a-CH_2 or 2´b-CH_2), 23.83 (9-CH_2), 23.18 (3´a-CH_2 or 3´b-CH_2), 23.12 (3´a-CH_2 or 3´b-CH_2), 14.58 (10-CH_3).

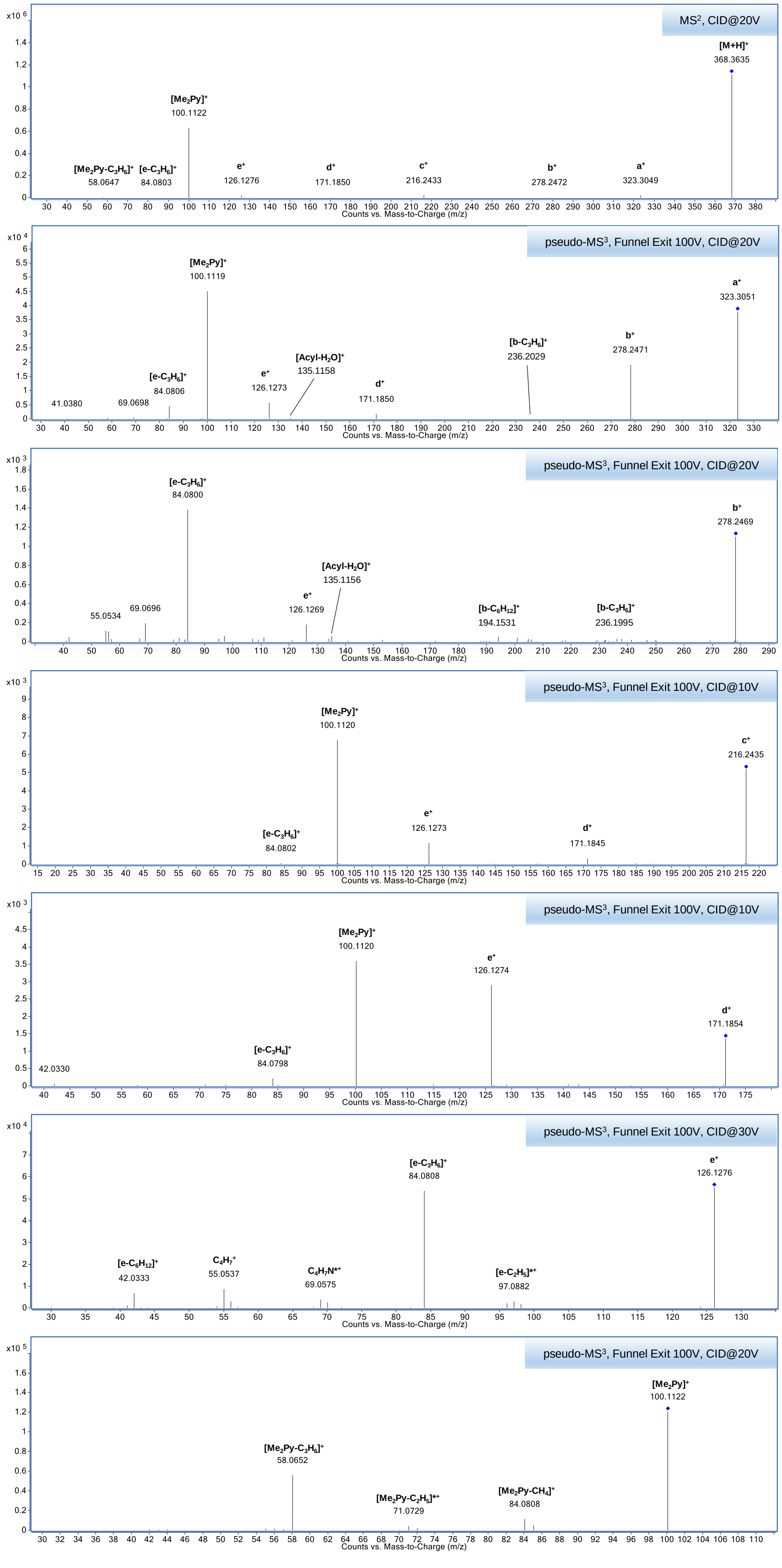

Figure S1. CID tandem mass spectra (MS^2 and pseudo-MS^3) of C10:1-solamine (**10a**). Spectra were obtained using UHPLC/ESI-QTOFMS in positive ion mode. For acquisition of pseudo-MS^3 spectra funnel exit voltage was increased from 50 V to 100 V. Precursor ions are marked with a blue diamond.

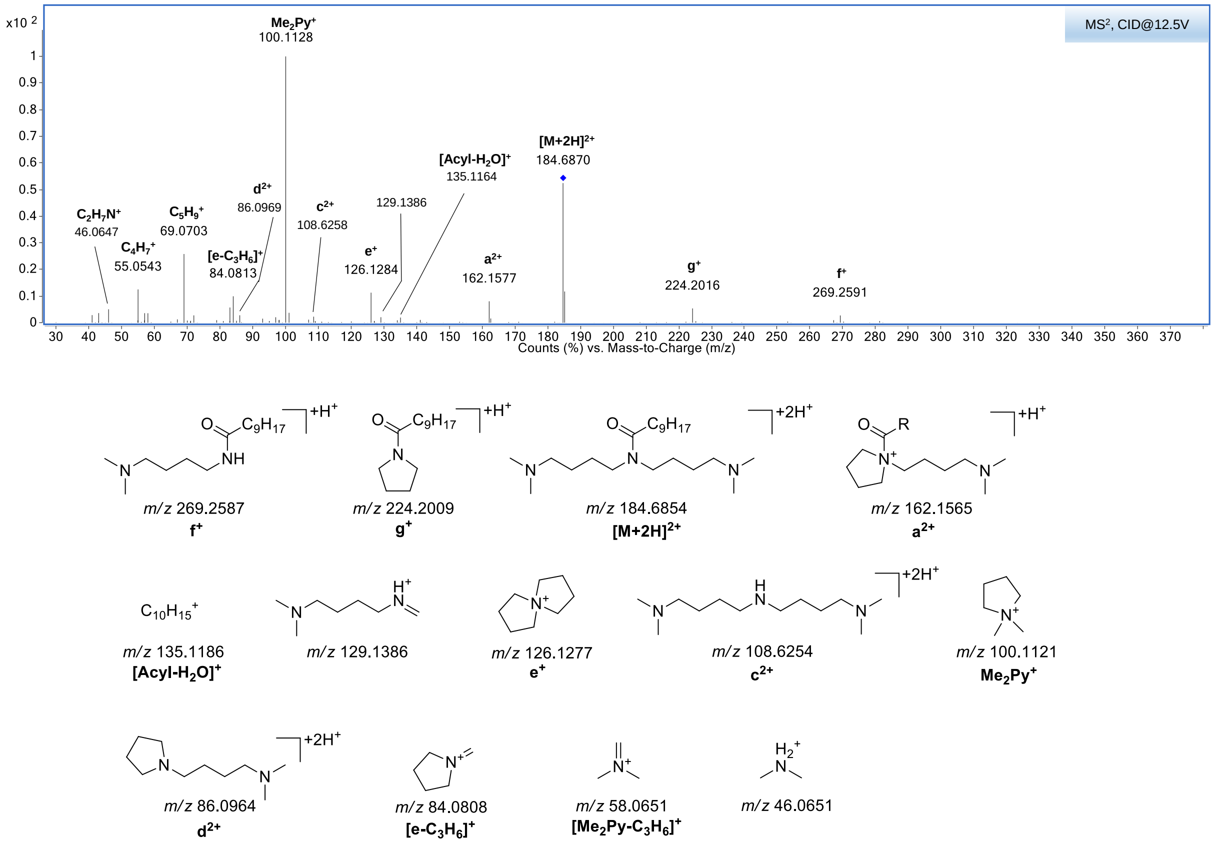

Figure S2. CID tandem mass spectrum of C10:1-solamine (**10a**) and putative structures with calculated *m/z* of the observed fragment ions. The spectrum was obtained from $[M+2H]^{2+}$ at a collision enery of 12.5 V using UHPLC/ESI-QTOFMS and a tuber periderm extract of *S. pinnatisectum* WKS 31607. The precursor ion is marked with a blue diamond.

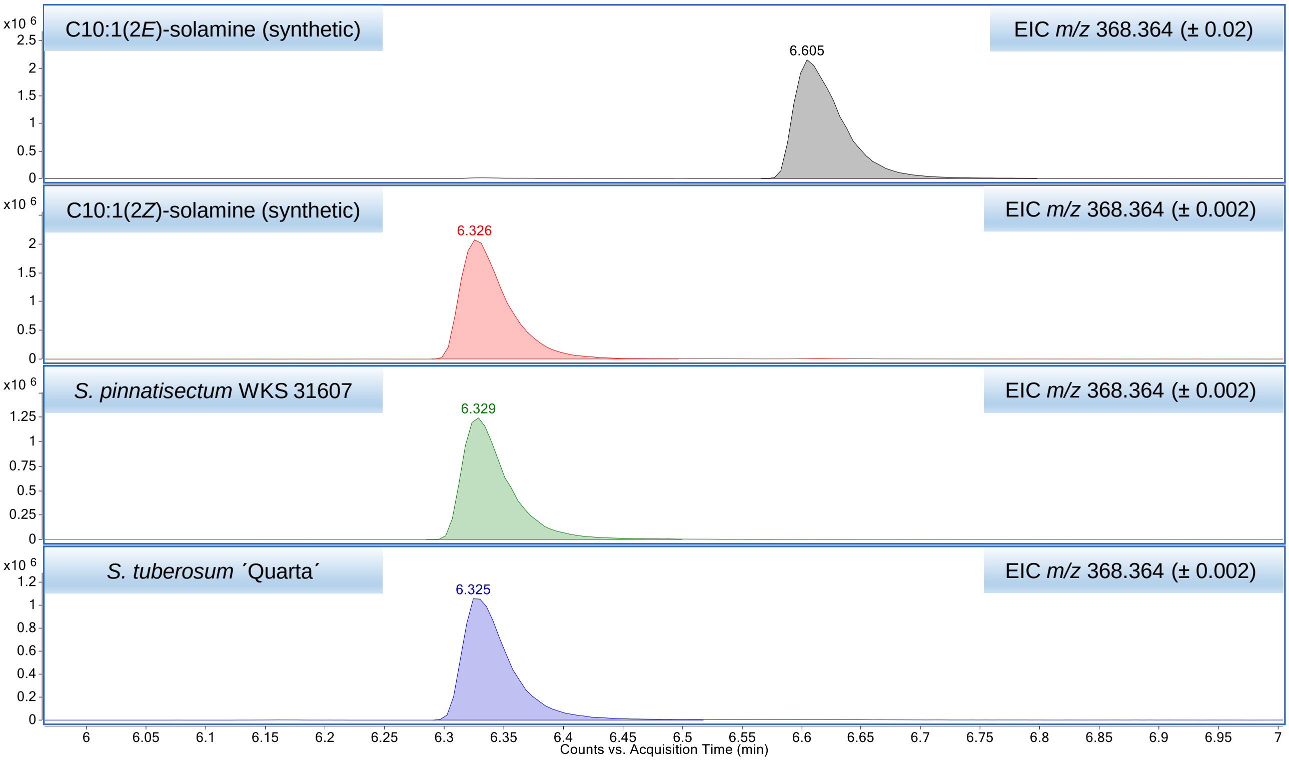

Figure S3. Authentication of C10:1-solamine (**10a**) detected in tuber periderm extracts of *S. pinnatisectum* and *S. tuberosum* using synthetic reference compounds C10:1(2*E*)-solamine dihydrochloride and C10:1(2*Z*)-solamine dihydrochloride.

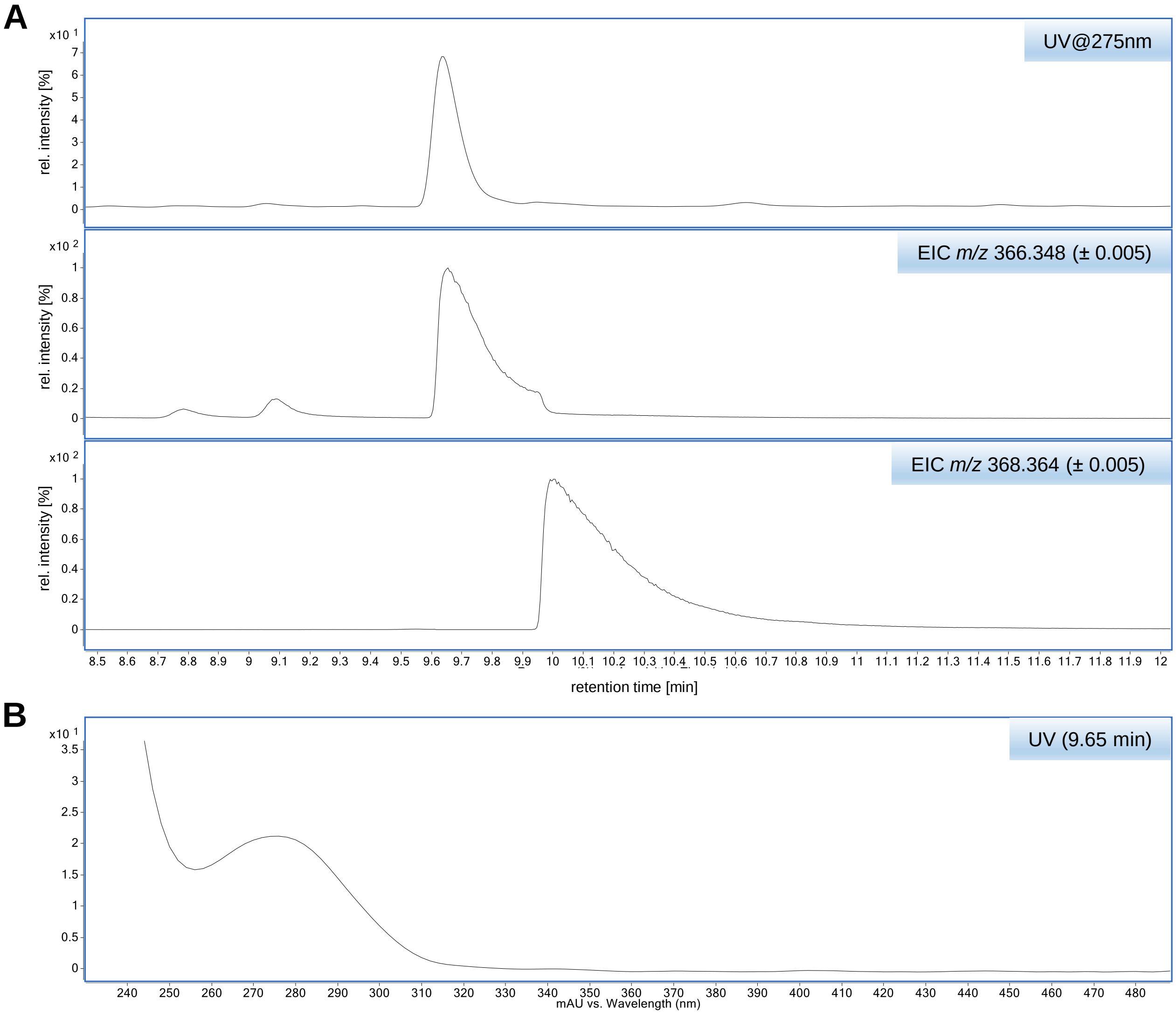

Figure S4. (A) Chromatographic separation of C10:1(2*E*)-solamine (**10a**) and C10:2-solamine-#2 (**12b**). Extracted ion chromatograms of $[M+H]^+$ and a UV chromatogram at 275 nm (bandwith 4 nm) are shown. Chromatograms were obtained using UHPLC/DAD/ESI-QTOFMS in positive ion mode. Chromatographic conditions were as follows: eluent A, 0.3% HCOOH in water; eluent B, 0.3% HCOOH in methanol; column, ACQUITY UPLC HSS T3 (2.1 mm × 100 mm, particle size 1.8 µm, pore size 100 Å), flow rate, 500 µL min^{-1}; column temperature, 40 °C; binary gradient program, 0 to 1 min, isocratic, 5% B; 1 to 15 min, linear gradient from 5% to 68% B; injection volume, 1 µL of the fraction obtained from a tuber periderm extract of *S. tubersoum* ´Quarta´ after cation exchange SPE (40 µg/µL) (B) Extracted UV spectrum of **12b**.

Table S1. Chromatographic peak characteristics of C16:0-solamine depending on column type, eluents, eluent additive and column temperature. Chromatograms were obtained using UHPLC/ESI-QTOFMS in positive ion mode. The following gradient program at a flow rate of 500 µL min^{-1} was used: 0 to 1 min, isocratic, 5% B; 1 to 19 min, linear gradient from 5% to 95% B. One µL of a solution of C16:0-solamine dihydrochloride in methanol (1 µM) was injected. Extracted ion chromatograms of $[M+H]^+$ of C16:0-solamine (*m/z* 454.473) were evaluated.

**column**	**eluent A**	**eluent B**	**eluent additive** *[v/v]*	**column temp.** *[°C]*	**C16:0-solamine**		
					t_R *[min]*	**width**[1] *[min]*	**tailing factor**[2]
A	H_2O	CH_3CN	0.1% HCO_2H	40	10.00	0.19	7.5
B	H_2O	CH_3CN	0.1% HCO_2H	40	9.63	0.46	10.5
A	H_2O	CH_3CN	0.5% HCO_2H	40	10.65	0.14	4.2
B	H_2O	CH_3CN	0.5% HCO_2H	40	10.22	0.23	6.3
A	H_2O	CH_3OH	0.5% HCO_2H	40	15.65	0.23	5.1
B	H_2O	CH_3OH	0.5% HCO_2H	40	15.14	0.28	5.8
A	H_2O	CH_3CN	0.5% CH_3CO_2H	40	9.46	0.33	4.4
A	H_2O	CH_3CN	0.5% HCO_2H	50	10.52	0.14	3.8

[1]peak width at 10% of peak height, [2]tailing factor at 10% of peak height

column A: ACQUITY UPLC HSS T3, 2.1 mm × 100 mm, particle size 1.8 µm, pore size 100 Å (Waters);

column B: Zorbax RRHD Eclipse Plus C18, 2.1 mm × 100 mm, particle size 1.8 µm, pore size 95 Å (Agilent Technologies)

Table S2. Analytical data of acyl solamines.

no.	name	elemental composition	*ret. time* [min]	$[M+H]^+$ det [*m/z*]	$[M+H]^+$ calc [*m/z*]	$[M+H]^{2+}$ det [*m/z*]	$[M+H]^{2+}$ calc [*m/z*]	CID mass spectra: *m/z* (relative intensity) [mass error in ppm] *CE* [V]	*precursor ion* $[M+H]^+$	*product ions* a^+	b^+	c^+	d^+	e^+	Me_2Py^+	$[e\text{-}C_3H_6]^+$	$[Me_2Py\text{-}C_3H_6]^+$	$[Acyl]^+$	other ions
								10	300.3008 (100) [-0.5]	255.2415 (0.3) [-6.2]	-	216.2426 (0.5) [-3.7]	-	-	100.1122 (5.5) [1.2]	84.0812 (0.1) [5.0]	58.0641 (0.2) [-17.7]	-	-
**1**	C5:0-Solamine	C17H37N3O	2.53	300.3011	300.3009	150.6544	150.6541	25	300.2993 (5.6) [-5.5]	255.2408 (1.4) [-9.0]	210.1848 (2.1) [-2.1]	216.2419 (0.6) [-7.0]	171.1859 (0.2) [1.9]	126.1276 (3.2) [-1.0]	100.1123 (100) [2.2]	84.0806 (1.6) [-2.1]	58.0649 (1) [-4.0]	85.0641 (0.3) [-8.1]	57.0690 (0.4) [-15.4] [Acyl-CO]+
								40	-	-	-	-	-	126.1280 (4) [2.1]	100.1126 (100) [5.2]	84.0809 (6.9) [1.4]	58.0647 (13.7) [-7.4]	85.0645 (1.1) [-3.4]	57.0699 (5.1) [0.4] [Acyl-CO]+
								10	314.3176 (100) [3.2]	269.2573 (0.3) [-5.3]	-	216.2436 (0.3) [0.8]	-	126.1272 (0.1) [-4.2]	100.1127 (4.1) [6.2]	-	-	-	-
**2a**	C6:0-Solamine	C18H39N3O	3.51	314.3166	314.3166	157.6623	157.6619	25	314.3171 (8) [1.6]	269.2586 (1.4) [-0.5]	224.2009 (1.6) [0]	216.2431 (0.4) [-1.5]	171.186 (0.3) [2.5]	126.1281 (3.2) [2.9]	100.1128 (100) [7.2]	84.0812 (0.9) [5.0]	58.0651 (0.9) [-0.5]	99.0808 (0.1) [3.6]	71.0853 (0.2) [-3.2] [Acyl-CO]+
								40	-	-	224.2000 (0.3) [-4]	-	-	126.1279 (3.9) [1.3]	100.1127 (100) [6.2]	84.0808 (5.3) [0.2]	58.0649 (10.2) [-4]	99.0805 (0.3) [0.6]	71.0853 (1.7) [-3.2] [Acyl-CO]+
								10	312.3014 (100) [1.5]	267.2433 (0.2) [0.8]	-	-	-	126.1291 (0.2) [10.9]	100.1123 (4.8) [2.2]	-	-	-	-
**3**	C6:1-Solamine	C18H37N3O	3.19	312.3013	312.3009	156.6545	156.6541	25	312.2996 (5) [-4.3]	267.2426 (1.3) [-1.8]	222.1843 (1.7) [-4.2]	-	-	126.1274 (1.9) [-2.6]	100.1124 (100) [3.2]	84.0810 (0.5) [2.6]	58.0644 (1.1) [-12.6]	97.0644 (1.7) [-4]	69.0692 (0.1) [-12.6] [Acyl-CO]+, 55.0173 (0.8) [-9.8] [C3H3O]+
								40	-	-	222.1857 (0.3) [2.1]	-	-	126.1271 (2.2) [-5.0]	100.1124 (100) [3.2]	84.0804 (2.8) [-4.5]	58.0648 (11.6) [-5.7]	97.0647 (4.8) [-0.9]	69.0690 (0.5) [-12.7] [Acyl-CO]+, 55.0176 (13.1) [-4.4] [C3H3O]+
								10	328.3331 (100) [2.6]	283.2728 (0.3) [-5.6]	-	216.2431 (0.3) [-1.5]	-	126.1276 (0.2) [-1.0]	100.1124 (5.1) [3.2]	-	-	-	-
**4**	C7:0-Solamine	C19H41N3O	4.43	328.3320	328.3322	164.6700	164.6698	25	328.3318 (9.9) [-1.3]	283.2733 (1.2) [-3.8]	238.2158 (1.4) [-3.1]	216.2427 (0.6) [-3.3]	171.1852 (0.3) [-2.2]	126.1275 (3.3) [-1.8]	100.1126 (100) [5.2]	84.0806 (0.9) [-2.1]	58.0646 (1) [-9.1]	113.0952 (0.1) [-7.9]	85.1000 (0.2) [-13.9] [Acyl-CO]+
								40	-	-	238.2163 (0.3) [-1.0]	-	-	126.1278 (4.3) [0.6]	100.1129 (100) [8.2]	84.0808 (4.6) [0.2]	58.0650 (9.3) [-2.2]	113.0954 (0.2) [-6.1]	85.1002 (0.7) [-11.5] [Acyl-CO]+
								10	342.3488 (100) [2.7]	297.2889 (0.2) [-3.8]	-	216.2436 (0.3) [0.8]	-	126.1273 (0.2) [-3.4]	100.1122 (5.5) [1.2]	-	-	-	-
**5**	C8:0-Solamine	C20H43N3O	5.33	342.3477	342.3479	171.6777	171.6776	25	342.3471 (13.6) [-2.3]	297.2889 (1.8) [-3.8]	252.2314 (1.3) [-3.1]	216.2429 (0.8) [-2.4]	171.1854 (0.3) [-1.1]	126.1277 (3.1) [-0.2]	100.1126 (100) [5.2]	84.0805 (0.9) [-3.3]	58.0647 (1) [-7.4]	-	-
								40	-	-	252.2311 (0.3) [-4.3]	-	-	126.1279 (4.2) [1.3]	100.1127 (100) [6.2]	84.0809 (4.1) [1.4]	58.0649 (8.5) [-4.0]	-	57.0698 (5.1) [-1.4] [C4H9]+
								10	340.3326 (100) [1.1]	295.2761 (0.3) [5.8]	-	216.2445 (0.3) [5]	-	126.1279 (0.4) [1.3]	100.1124 (6.7) [3.2]	-	-	-	-
**6a**	C8:1-Solamine-#1	C20H41N3O	4.70	340.3318	340.3322	170.6698	170.6698	25	340.3309 (10) [-3.9]	295.2724 (1.2) [-6.7]	250.2155 (1.1) [-4.2]	216.2421 (0.7) [-6.1]	171.1857 (0.3) [0.7]	126.1271 (3.6) [-5.0]	100.1124 (100) [3.2]	84.0808 (0.7) [0.2]	58.0647 (1.3) [-7.4]	-	55.0539 (0.5) [-6.0] [C4H7]+
								40	-	-	250.2156 (0.3) [-3.8]	-	-	126.1272 (4.4) [-4.2]	100.1124 (100) [3.2]	84.0808 (4.6) [0.2]	58.0648 (9.1) [-5.7]	-	55.0538 (5.2) [-7.8] [C4H7]+
								10	340.3328 (100) [1.6]	295.2737 (0.3) [-2.3]	-	-	-	126.1264 (0.2) [-10.5]	100.1121 (6.3) [0.2]	-	-	-	-
**6b**	C8:1-Solamine-#2	C20H41N3O	5.01	340.3319	340.3322	170.6700	170.6698	25	340.3309 (9) [-3.9]	295.2737 (1.6) [-2.3]	250.2157 (1.3) [-3.4]	-	171.1844 (0.1) [-6.9]	126.1274 (2.0) [-2.6]	100.1124 (100) [3.2]	84.0807 (0.3) [-1]	58.0647 (1.1) [-7.4]	125.0960 (1.1) [-0.7]	97.1009 (0.2) [-2.9] [Acyl-CO]+, 55.0538 (0.5) [-7.8] [C4H7]+
								40	-	-	250.2151 (0.4) [-5.8]	-	-	126.1275 (2.8) [-1.8]	100.1126 (100) [5.2]	84.0808 (2.3) [0.2]	58.0648 (8.3) [-5.7]	125.0959 (1.7) [-1.5]	97.1016 (0.4) [4.3] [Acyl-CO]+, 55.0541 (4.7) [-2.4] [C4H7]+, 55.0177 (9.5) [-2.5] [C3H3O]+
								10	338.3170 (100) [1.2]	293.2569 (0.5) [-6.3]	248.1982 (0.1) [-10.8]	216.2449 (0.2) [6.8]	-	126.1275 (0.3) [-1.8]	100.1121 (6.9) [0.2]	-	-	123.0792 (0.3) [-10.1]	95.0856 (0.1) [0.7] [Acyl-CO]+
**7**	C8:2-Solamine	C20H39N3O	4.75	338.3166	338.3166	169.6622	169.6619	25	338.3152 (6.1) [-4.1]	293.2571 (1.4) [-5.6]	248.2011 (0.8) [0.8]	216.2427 (0.5) [-3.3]	171.1847 (0.3) [-5.1]	126.1276 (3.3) [-1.0]	100.1125 (100) [4.2]	84.0814 (0.3) [7.4]	58.0648 (0.9) [-5.7]	123.0803 (8.5) [-1.1]	95.0854 (4.0) [-1.4] [Acyl-CO]+, 81.0334 (2.1) [-1.1] [C5H5O]+, 53.0381 (0.5) [-9.1] (C4H5]+
								40	-	-	-	-	-	126.1279 (5.0) [1.3]	100.1127 (100) [6.2]	84.0808 (2.2) [0.2]	58.0648 (7.6) [-5.7]	123.0807 (5.6) [2.1]	95.0856 (14.8) [0.7] [Acyl-CO]+, 81.0334 (22.1) [2.6] [C5H5O]+, 67.0540 (5.6) [-3.4] [C5H7]+, 55.0542 (1.7) [-0.5] [C4H7]+, 55.0177 (1.1) [-2.5] [C3H3O]+. 53.0381 (7.8) [-1.5] (C4H5]+
								10	356.3643 (100) [2.1]	311.3027 (0.2) [-9.6]	-	216.2418 (0.2) [-7.5]	-	126.1270 (0.2) [-5.8]	100.1121 (4.5) [0.2]	-	-	-	-
**8**	C9:0-Solamine	C21H45N3O	6.17	356.3637	356.3635	178.6857	178.6854	25	356.3625 (18.2) [-2.9]	311.3036 (1.6) [-6.7]	266.2460 (1.2) [-6.9]	216.2422 (1.4) [-5.6]	171.1848 (0.7) [-4.6]	126.1275 (3.6) [-1.8]	100.1124 (100) [3.2]	84.0807 (0.6) [-1]	58.0649 (1.3) [-4]	-	71.0848 (0.3) [-10.3] [C5H11]+, 57.0700 (0.3)[2.1] [C4H9]+
								40	-	-	266.2457 (0.3) [-8]	-	-	126.1272 (5.4) [-4.2]	100.1125 (100) [4.2]	84.0807 (4.4) [-1]	58.0646 (6.9) [-9.1]	-	71.0852 (1.4) [-4.6] [C5H11]+, 57.0696 (2.5)[-4.9] [C4H9]+
								10	370.3797 (100) [1.4]	325.3182 (0.2) [-9.7]	-	216.2440 (0.2) [2.7]	-	126.1270 (0.2) [-5.8]	100.1120 (4.4) [-0.8]	-	-	-	-
**9**	C10:0-Solamine	C22H47N3O	6.94	370.3790	370.3792	185.6933	185.6932	25	370.3791 (27.9) [-0.2]	325.3201 (1.6) [-3.8]	280.2625 (1.0) [-3.5]	216.2424 (1.2) [-4.7]	171.1851 (0.5) [-2.8]	126.1273 (3.6) [-3.4]	100.1126 (100) [5.2]	84.0806 (0.6) [-2.1]	58.0646 (0.9) [-9.1]	-	71.0848 (0.2) [-10.3] [C5H11]+, 57.0692 (0.3)[-11.9] [C4H9]+
								40	-	-	280.2626 (0.4) [-3.2]	-	-	126.1277 (4.6) [-0.2]	100.1129 (100) [8.2]	84.0808 (3.3) [0.2]	58.0649 (5.9) [-4]	-	71.0854 (1.0) [-1.8] [C5H11]+, 57.0698 (0.9)[-1.4] [C4H9]+
								10	368.3643 (100) [2.1]	323.3041 (0.1) [-4.9]	-	216.2428 (0.2) [-2.9]	-	126.1281 (0.1) [2.9]	100.1126 (5.3) [5.2]	-	-	-	-

**10a**	C10:1-Solamine	C22H45N3O	6.36	368.3634	368.3635	184.6855	184.6854	25	368.3644 (20.8) [2.3]	323.3051 (1.4) [-1.8]	278.2474 (0.9) [-1.6]	216.2432 (0.9) [-1]	171.1855 (0.3) [-0.5]	126.1280 (3.1) [2.1]	100.1127 (100) [6.2]	84.0809 (0.6) [1.4]	58.0648 (0.9) [-5.7]	-	69.0697 (0.2) [-2.6] [C5H9]+, 55.0537 (0.1)[-9.6] [C4H7]+
								40	-	-	278.2470 (0.2) [-3.0]	-	-	126.1281 (4.4) [2.9]	100.1129 (100) [8.2]	84.0811 (2.9) [3.8]	58.0652 (6.5) [1.2]	-	69.0699 (1.7) [0.3] [C5H9]+, 55.0541 (1.0)[-2.4] [C4H7]+
**11***	C10:1-O1-Solamine	C22H45N3O2	3.8-5.4	384.3581	384.3585	192.6830	192.6829	10	384.3591 (100) [1.7]	339.2986 (0.1) [-5.9]	-	216.2415 (0.2) [-8.9]	-	126.1265 (0.2) [-9.8]	100.1122 (3.9) [1.2]	-	-	-	-
								25	384.3583 (38.2) [-0.4]	339.2999 (1.8) [-2.1]	294.2412 (0.7) [-5.3]	216.2426 (1.6) [-3.8]	171.1857 (0.5) [0.7]	126.1273 (3) [-3.4]	100.1125 (100) [4.2]	84.0802 (0.6) [-6.9]	58.0647 (1) [-7.4]	-	-
								40	-	-	294.2419 (0.4) [-2.9]	-	-	126.1275 (4.4) [-1.8]	100.1127 (100) [6.2]	84.0809 (2.4) [1.4]	58.0649 (5.2) [-4.0]		81.0697 (0.8) [-2.2] [C6H9]+, 67.0540 (0.6)[-3.4] [C5H7]+
**12a**	C10:2-Solamine-#1	C22H43N3O	5.69	366.3478	366.3479	183.6776	183.6776	10	366.3488 (100) [2.5]	321.2890 (0.2) [-3.2]	-	216.2432 (0.2) [-1.0]	-	126.1276 (0.2) [-1.0]	100.1125 (5.6) [4.2]	-	-	-	-
								25	366.3482 (17.3) [0.8]	321.2890 (1.2) [-3.2]	276.2315 (0.9) [-2.5]	216.2428 (0.8) [-2.9]	171.1853 (0.3) [-1.6]	126.1276 (3.1) [-1]	100.1125 (100) [4.2]	84.0806 (0.6) [-2.1]	58.0649 (0.9) [-4.0]	-	-
								40	-	-	276.2318 (0.2) [-1.4]	-	-	126.1279 (4.2) [1.3]	100.1128 (100) [7.2]	84.0809 (3) [1.4]	58.0650 (6.9) [-2.2]	-	81.0697 (0.7) [-2.2] [C6H9]+, 67.0540 (0.9) [-3.4] [C5H7]+, 55.0540 (0.6) [-4.2] [C4H7]+
**12b**	C10:2-Solamine-#2	C22H43N3O	6.33	366.3479	366.3479	183.6779	183.6776	10	366.3485 (100) [1.7]	321.2881 (0.2) [-6]	-	216.2428 (0.1) [-2.9]	-	126.1269 (0.2) [-6.6]	100.1123 (5.9) [2.2]	-	-	-	-
								25	366.3478 (15.6) [-0.2]	321.2888 (1.3) [-3.9]	276.2308 (0.7) [-5.0]	216.2427 (0.8) [-3.3]	171.1848 (0.5) [-4.6]	126.1274 (3.3) [-2.6]	100.1126 (100) [5.2]	84.0805 (0.3) [-3.3]	58.0648 (0.7) [-5.7]	151.1116 (2.0) [-0.9]	133.1003 (0.3) [-6.6] [Acyl-CO]+, 81.0336 (1.9) [1.4] [C5H5O]+, 69.0696 (0.9) [-4.1] [C5H9]+, 67.0537 (0.5) [-7.9] [C5H7]+, 53.0381 (0.4) [-9.1] [C4H5]+
								40	-	-	276.2300 (0.2) [-7.9]	-	-	126.1277 (4.5) [-0.2]	100.1128 (100) [7.2]	84.0809 (1.7) [1.4]	58.0650 (5.1) [-2.2]	151.1116 (0.8) [-0.9]	133.1007 (0.4) [-3.6] [Acyl-CO]+, 95.0494 (2.7) [2.7] [C6H7O]+, 81.0338 (13.8) [3.8] [C5H5O]+, 69.0698 (4.6) [-1.2] [C5H9]+, 53.0381 (4.7) [-3.4] [C4H5]+
**13***	C10:2-O1-Solamine	C22H43N3O2	3.6-5.2	382.3424	382.3428	191.6751	191.6750	10	382.3430 (100) [0.5]	-	-	216.2423 (0.8) [-5.2]		126.1268 (0.5) [-7.4]	100.1120 (8.9) [-0.8]	-	-	-	364.3315 (20.6) [-2.0] [M+H-H2O]+
								25	382.3410 (1.3) [-4.7]	-	-	216.2428 (3.5) [-2.9]	171.1850 (1.0) [-3.4]	126.1275 (5.3) [-1.8]	100.1123 (100) [2.2]	84.0809 (0.4) [1.4]	58.0645 (0.9) [-10.8]	-	364.3314 (17.6) [-2.3] [M+H-H2O]+, 319.2727 (0.8) [-5.3] [M+H-H2O-C2H7N]+, 274.2156 (0.3) [-3.4] [M+H-H2O-2C2H7N]+
								40	-	-	-	-	-	126.1274 (6.4) [-2.6]	100.1125 (100) [4.2]	84.0806 (2.0) [-2.1]	58.0645 (5.1) [-10.8]	-	274.2160 (0.2) [-2.0] [M+H-H2O-2C2H7N]+, 93.0695 (0.7) [-4.1] [C7H9]+, 79.0538 (1.0) [-10.8] [C6H7]+
**14**	C11:1-Solamine	C23H47N3O	6.82	382.3793	382.3792	191.6936	191.6932	10	382.3798 (100) [1.6]	-	-	216.2416 (0.3) [-8.4]	171.1854 (0.1) [-1.1]	126.1272 (0.2) [-4.2]	100.1122 (4.6) [1.2]	-	-	-	-
								25	382.3784 (25.3) [-2.1]	337.3187 (0.9) [-7.8]	292.2610 (0.6) [-8.5]	216.2428 (2.0) [-2.9]	171.1858 (0.6) [1.3]	126.1276 (3.5) [-1.0]	100.1125 (100) [4.2]	84.0807 (0.6) [-1]	58.0648 (1.1) [-5.7]	-	-
								40	-	-	-	-	-	126.1274 (3.8) [-2.6]	100.1125 (100) [4.2]	84.0806 (2.3) [-2.1]	58.0648 (5.9) [-5.7]	-	83.0849 (0.7) [-7.6] [C6H11]+, 69.0694 (0.7) [-6.9] [C5H9]+, 55.0537 (1.7) [-9.6] [C4H7]+
**15**	Benzoyl-solamine	C19H33N3O	1.89	320.2692	320.2696	160.6386	160.6385	10	320.2697 (100) [0.2]	-	-	-	-	-	100.1120 (11.2) [-0.8]	-	-	-	-
								25	320.2681 (3.2) [-4.8]	275.2099 (0.5) [-6.9]	230.1529 (1) [-4.5]	-	-	126.1272 (0.5) [-4.2]	100.1123 (100) [2.2]	84.0802 (0.3) [-6.9]	58.0648 (1.1) [-5.7]	105.0332 (3.8) [-2.8]	77.0381 (0.2) [-6.2] [Acyl-CO]+
								40	-	-	230.1526 (0.2) [-5.8]	-	-	126.1288 (0.5) [8.5]	100.1123 (100) [2.2]	84.0804 (3.2) [-4.5]	58.0645 (14.6) [-10.8]	105.0333 (21.0) [-1.8]	77.0386 (5.7) [0.3] [Acyl-CO]+
**16**	Dihydroferuloyl-solamine	C22H39N3O3	2.83	394.3060	394.3065	197.6567	197.6569	10	394.3059 (100) [-1.3]	-	-	216.2408 (0.2) [-12.1]	-	126.1250 (0.3) [-21.6]	100.1120 (5.9) [-0.8]	-	-	-	-
								25	394.3054 (30.3) [-2.6]	349.2465 (1.9) [-5.9]	304.1878 (0.9) [-9.6]	216.242 (2.2) [-6.6]	171.1857 (0.6) [0.7]	126.1279 (3.9) [1.3]	100.1122 (100) [1.2]	84.0799 (0.7) [-10.5]	58.0648 (0.8) [-5.7]	-	137.0599 (1.1) [1.4] [Acyl-C2H2O]+
								40	-	-	304.1888 (0.3) [-6.3]	-	-	126.1275 (4.3) [-1.8]	100.1124 (100) [3.2]	84.0806 (2.5) [-2.1]	58.0645 (7.0) [-10.8]	-	137.0592 (7.4) [-3.7] [Acyl-C2H2O]+
**17a/b****	Cinnamoyl-solamine	C21H35N3O	3.18/3.89	346.2846	346.2853	173.6461	173.6463	10	346.2836 (100) [-4.9]	-	-	-	-	-	100.1120 (7.2) [-0.8]	-	-	131.0488 (1.3) [-2.6]	-
								25	346.2836 (8.2) [-4.9]	301.2246 (0.9) [-9.4]	256.1689 (0.5) [-2.7]	216.2421 (0.9) [-6.1]	171.1851 (0.6) [-2.8]	126.1266 (5.3) [-9.0]	100.1120 (100) [-0.8]	84.0806 (0.5) [-2.1]	58.0647 (1.1) [-7.4]	131.0488 (12.8) [-2.6]	103.0533 (1.5) [-9.0] [Acyl-CO]+
								40	-	-	-	-	-	126.1271 (9.1) [-5.0]	100.1121 (100) [0.2]	84.0806 (2.7) [-2.1]	58.0644 (6.6) [-12.6]	131.049 (33.7) [-1.1]	103.0538 (21.2) [-4.2] [Acyl-CO]+, 77.0378 (1.7) [-10.1] [C6H5]+
**18a/b****	Coumaroyl-solamine	C21H35N3O2	2.12/2.85	362.2800	362.2802	181.6437	181.6437	10	362.2790 (100) [-3.3]	317.2221 (0.5) [-0.8]	-	216.2401 (0.6) [-15.4]	171.1839 (0.2) [-9.8]	126.1291 (0.3) [10.9]	100.1117 (7.3) [-3.8]	-	-	147.0427 (1.4) [-9.2]	-
								25	362.2783 (11.5) [-5.2]	317.2199 (1.5) [-7.7]	272.1629 (0.3) [-5.9]	216.2422 (3) [-5.6]	171.1861 (1.0) [3.0]	126.1270 (5.5) [-5.8]	100.1121 (100) [0.2]	84.0803 (0.7) [-5.7]	58.0648 (0.7) [-5.7]	147.0442 (39.2) [1]	119.0480 (1.9) [-9.6] [Acyl-CO]+, 91.0538 (0.9) [-4.7] [Acyl-2CO]+
								40	-	-	-	-	-	126.1276 (8.8) [-1]	100.1120 (100) [-0.8]	84.0810 (1.8) [2.6]	58.0651 (4.1) [-0.5]	147.0439 (93) [-1.1]	119.0489 (24.9) [-2.9] [Acyl-CO]+, 91.0539 (11.0) [-3.6] [Acyl-2CO]+
**19a/b****	Caffeoyl-solamine	C21H35N3O3	1.42/2.38	378.2748	378.2751	189.6414	189.6412	10	378.2750 (100) [-0.3]	333.2161 (0.3) [-3.5]	-	216.2432 (0.5) [-1.0]	171.1841 (0.2) [-8.6]	126.1265 (0.3) [-9.8]	100.1122 (5.8) [1.2]	-	-	163.0383 (1.0) [-4.1]	-
								25	378.2751 (16.8) [-0.1]	333.2157 (1.1) [-4.7]	288.1578 (0.3) [-5.6]	216.2430 (2.8) [-1.9]	171.1852 (1.2) [-2.2]	126.1276 (5.6) [-1.0]	100.1125 (100) [4.2]	84.0806 (0.2) [-2.1]	58.0646 (0.5) [-9.1]	163.0394 (28.2) [2.6]	145.0280 (0.9) [-2.8] [Acyl-H2O]+, 135.0431 (0.6) [-7.1] [Acyl-CO]+, 117.0335 (0.4) [0.1] [Acyl-H2O-CO]+

								40	-	-	-	-	-	126.1277 (8.6) [-0.2]	100.1125 (100) [4.2]	84.0806 (1.3) [-2.1]	58.0647 (4.3) [-7.4]	163.0396 (63.8) [3.9]	145.0284 (14.6) [-0.1] [Acyl-H2O]+, 135.0441 (11.5) [0.3] [Acyl-CO]+, 117.0336 (6.1) [0.9] [Acyl-H2O-CO]+, 89.0384 (3.0) [-2.0] [Acyl-H2O-2CO]+
**20a/b****	Feruloyl-solamine	C22H37N3O3	2.53/3.13	392.2905	392.2908	196.6493	196.6490	10	392.2912 (100) [1.1]	347.2324 (0.2) [-1.5]	-	216.2430 (0.6) [-1.9]	-	126.1269 (0.2) [-6.6]	100.1124 (4.5) [3.2]	-	-	177.0548 (1.6) [1.0]	145.0281 (0.3) [-2.1] [Acyl-CH3OH]+
								25	392.2900 (19.8) [-2.0]	347.2315 (1.3) [-4.1]	302.1717 (0.2) [-11.2]	216.2429 (6.1) [-2.4]	171.1849 (1.5) [-4.0]	126.1277 (4.9) [-0.2]	100.1126 (100) [5.2]	84.0811 (0.1) [3.8]	58.0644 (0.6) [-12.6]	177.0552 (70) [3.3]	149.0587 (0.4) [-6.8] [Acyl-CO]+, 145.0284 (7.3) [-0.1] [Acyl-CH3OH]+, 117.0334 (1.1) [-0.8] [Acyl-CH3OH-CO]+
								40	-	-	-	-	-	126.1275 (8.0) [-1.8]	100.1126 (100) [5.2]	84.0808 (1.0) [0.2]	58.0649 (3.5) [-4.0]	177.0551 (84.2) [2.7]	162.0313 (0.3) [0.9] [Acyl-CH3]*+, 149.0596 (6.9) [-0.7] [Acyl-CO]+, 145.0290 (93) [4.1] [Acyl-CH3OH]+, 134.0360 (1.0) [-1.7] [Acyl-CO-CH3]*+, 117.0337 (19.6) [1.8] [Acyl-CH3OH-CO]+, 89.0385 (4.2) [-0.9] [Acyl-CH3OH-2CO]+

* Up to 10 isomeric components with similar CID mass spectra. Reported mass spectral data were obtained from the most abundant isomer.

** Pairs of E/Z isomers with similar CID mass spectra. Reported mass spectral data were obtained from the later eluting isomer.

Table S3. Analytical data of fatty acyl solamine N-oxides.

								CID mass spectra: m/z (relative intensity) [mass error in ppm]										
no.	name	elemental composition	*ret. time* [min]	$[M+H]^+$ det [m/z]	$[M+H]^+$ calc [m/z]	$[M+H]^{2+}$ det [m/z]	$[M+H]^{2+}$ calc [m/z]	*CE* [V]	*precursor ion* $[M+H]^+$	a^+	b^+	d^+	e^+	Me_2Py^+	$[e-C_3H_6]^+$	$[Me_2Py-C_3H_6]^+$	$Acyl^+$	other ions
**2b**	C6:0-solamine-*N*-oxide	C18H39N3O2	3.79	330.31137	330.31150	165.65986	165.65939	10	330.3118 (86.4) [0.9]	269.2594 (100) [2.5]	224.2003 (0.7) [-2.6]	171.1850 (0.4) [-3.4]	126.1274 (0.4) [-2.6]	100.1120 (3.5) [-0.8]	84.0808 (0.2) [0.2]	58.0643 (0.4) [-14.3]	-	-
								25	330.3114 (0.1) [-0.3]	269.2594 (90.7) [2.5]	224.2009 (39.2) [0.0]	171.1854 (3.6) [-1.1]	126.1277 (9.3) [-0.2]	100.1125 (100) [4.2]	84.0806 (11.2) [-2.1]	58.0648 (7.5) [-5.7]	99.0804 (0.8) [-0.4]	71.0849 (1.0) [-8.9] [Acyl-CO]+
								40	-	269.2576 (0.5) [-4.2]	224.2001 (5.9) [-3.5]	-	126.1279 (6.4) [1.3]	100.1123 (100) [2.2]	84.0806 (36.9) [-2.1]	58.0646 (16.1) [-9.1]	99.0800 (2.9) [-4.4]	71.0851 (6.5) [-6.0] [Acyl-CO]+
**10b**	C10:1-solamine-*N*-oxide	C22H45N3O2	6.54	384.3580	384.3585	192.6831	192.6829	10	384.3606 (100) [5.6]	323.3074 (72.8) [5.3]	278.2486 (0.7) [2.7]	171.185 (0.3) [-3.4]	126.1271 (0.4) [-5.0]	100.112 (3.8) [-0.8]	84.0804 (0.3) [-4.5]	58.0644 (0.2) [-12.6]	-	-
								25	384.3594 (0.3) [2.5]	323.3070 (100) [4.1]	278.2482 (18.5) [1.3]	171.1855 (2.2) [-0.5]	126.1276 (6.1) [-1.0]	100.1124 (63.4) [3.2]	84.0805 (4.0) [-3.3]	58.0645 (4.3) [-10.8]	-	-
								40	-	323.3049 (1.0) [-2.4]	278.2472 (7.1) [-2.3]	-	126.1273 (7.5) [-3.4]	100.1122 (100) [1.2]	84.0806 (26) [-2.1]	58.0644 (11.6) [-12.6]	-	135.1154 (0.9) [-10.6] [Acyl-H2O]+, 83.0851 (1.0) [-5.2] [C6H11+], 69.0693 (4.2) [-8.4] [C5H9]+

Table S4. Analytical data of fatty acyl solamine di-N-oxides.

								CID mass spectra: *m/z* (relative intensity) [mass error in ppm]								
no.	name	elemental composition	*ret. time*	$[M+H]^+$ det	$[M+H]^+$ calc	$[M+H]^{2+}$ det	$[M+H]^{2+}$ calc	*CE*	*precursor ion*							
			[min]	[m/z]	[m/z]	[m/z]	[m/z]	[V]	$[M+H]^+$	a^+	b^+	$[b-C_3H_6]^+$	e^+	$[e-C_3H_6]^+$	$Acyl^+$	other ions
**2c**	C6:0-solamine-di-*N*-oxide	C18H39N3O3	4.02	346.3058	346.3064	173.6575	173.6568	10	346.3058 (100) [-1.8]	285.2526 (39.5) [-3.7]	224.2002 (4.7) [-3.1]	-	126.1262 (0.7) [-12.1]	84.0797 (0.5) [-12.8]	-	-
				346.3062	346.3064	173.6573	173.6569	25	346.3062 (0.3) [-0.7]	285.2528 (37.1) [-3.0]	224.2005 (100) [-1.7]	182.1545 (0.8) [3.1]	126.1275 (8.5) [-1.8]	84.0805 (13) [-3.3]	99.0799 (0.8) [-5.5]	100.1119 (1.9) [-1.8] [C6H14N]+, 71.0844 (0.4) [-15.9] [Acyl-CO]+, 58.0642 (2.6) [-16] [C3H8N+]
				-0.5		2.4		40	-	-	224.2001 (34.9) [-3.5]	182.1531 (1.2) [-4.6]	126.1272 (10.9) [-4.2]	84.0806 (100) [-2.1]	99.0802 (7.9) [-2.4]	100.1112 (3.4) [-8.8] [C6H14N+], 71.0853 (10.8) [-3.2] [Acyl-CO]+, 58.0646 (5.8) [-9.1] [C3H8N]+, 56.0488 (6.3) [-12.1] [C3H6N]+, 55.0536 (4.8) [-11.4] [C4H7]+
**10c**	C10:1-solamine-di-*N*-oxide	C22H45N3O3	6.65	400.3541	400.3534	200.6812	200.6803	10	400.3548 (100) [3.6]	339.3008 (21.4) [-1.0]	278.2472 (2.5) [-2.3]	-	126.1274 (0.4) [-2.6]	84.0800 (0.3) [-9.3]	-	-
				400.3530	400.3534	200.6805	200.6803	25	400.3525 (1.2) [-2.2]	339.3014 (73.6) [0.7]	278.2487 (100) [3.1]	236.1990 (0.2) [-8.0]	126.1280 (9.4) [2.1]	84.0805 (8.5) [-3.3]	-	135.1168 (0.5) [-0.2] [Acyl-H2O]+, 100.1117 (2.6) [-3.8] [C6H14N]+, 58.0646 (2.4) [-9.1] [C3H8N]+
				-0.9		0.6		40	-	-	278.2482 (62.4) [1.3]	236.2000 (0.9) [-3.8]	126.1276 (14.4) [-1.0]	84.0808 (100) [0.2]	-	135.1164 (3.4) [-3.2] [Acyl-H2O]+, 100.1118 (2.9) [-2.8] [C6H14N]+, 83.0854 (2.5) [-1.6] [C6H11]+, 69.0694 (8.4) [-6.9] [C5H9]+, 58.0646 (7.7) [-9.1] [C3H8N]+, 56.0488 (3.9) [-12.1] [C3H6N]+, 55.0538 (6.4) [-7.8] [C4H7]+

Table S5. Analytical data of acyl nor-solamines.

no.	name	elemental composition	*ret. time* [min]	$[M+H]^+$ det [m/z]	$[M+H]^+$ calc [m/z]	$[M+H]^{2+}$ det [m/z]	$[M+H]^{2+}$ calc [m/z]	*CE* [V]	CID mass spectra: *m/z* (relative intensity) [mass error in ppm] — *precursor ion* $[M+H]^+$	a^+	b^+	f^+	g^+	c^+	d^+	e^+	Me_2Py+	$[MePy+H]^+$	$[e-C_3H_6]^+$	$[Me_2Py-C_3H_6]^+$	$Acyl^+$	other ions
**2d**	C6:0-norsolamine	C17H37N3O	3.42	300.3015	300.3009	150.6545	150.6541	10	300.3016 (100) [2.2]	255.2413 (0.5) [-7.0]	-	215.2122 (0.1) [1.9]	170.1533 (0.5) [-3.8]	202.2278 (0.4) [0.1]	-	126.1284 (0.2) [5.3]	100.1124 (3.0) [3.2]	86.0961 (1.8) [-3.8]	-	-	-	-
								25	300.3000 (7.6) [-3.1]	255.2414 (2.2) [-6.6]	224.2012 (2.5) [1.4]	215.2133 (0.6) [7.0]	170.1538 (10.2) [-0.8]	202.2263 (0.8) [-7.3]	157.1694 (0.8) [-3.4]	126.1276 (6.6) [-1.0]	100.1123 (100) [2.2]	86.0964 (75.4) [-0.3]	84.0805 (3.0) [-3.3]	58.0643 (1.3) [-14.3]	99.0805 (1.2) [0.6]	71.0848 (1.6) [-10.3] [Acyl-CO]+
								40	-	-	224.199 (0.5) [-8.4]	-	170.1536 (0.7) [-2.0]	-	-	126.1279 (8.3) [1.3]	100.1125 (100) [4.2]	86.0967 (94.4) [3.1]	84.0808 (9.6) [0.2]	58.0647 (14.4) [-7.4]	99.0801 (1.9) [-3.4]	71.0855 (6.2) [-0.4] [Acyl-CO]+
**10d**	C10:1-norsolamine	C21H43N3O	6.29	354.3477	354.3479	177.6778	177.6776	10	354.3488 (100) [2.6]	309.2885 (0.4) [-5.0]	-	269.2579 (0.1) [-3.1]	224.2006 (0.5) [-1.3]	202.2277 (0.3) [-0.3]	-	126.1276 (0.1) [-1.0]	100.1125 (3.3) [4.2]	86.0965 (1.6) [0.8]	-	-	-	-
								58	354.3481 (21.4) [0.6]	309.2892 (2.8) [-2.7]	278.2469 (1.4) [-3.4]	269.2582 (1) [-2.0]	224.2011 (10.1) [0.9]	202.2275 (1.4) [-1.3]	157.1696 (0.8) [-2.1]	126.1278 (5.8) [0.6]	100.1128 (100) [7.2]	86.0969 (58.6) [5.5]	84.0809 (1.2) [1.4]	58.0649 (1) [-4.0]	153.1271 (0.1) [-1.9]	135.1167 (0.4) [-1.0] [Acyl-H2O]+, 69.0699 (0.6) [0.3] [C5H9]+
								40	-	-	-	-	224.2009 (0.7) [0.0]	-	-	126.1281 (8.0) [2.9]	100.1129 (100) [8.2]	86.0972 (77.4) [8.9]	84.0811 (5.1) [3.8]	58.0651 (8.4) [-0.5]	-	135.1168 (0.7) [-0.2] [Acyl-H2O]+, 83.0855 (0.8) [-0.4] [C6H11]+, 69.0699 (5.1) [0.3] [C5H9]+, 55.0543 (2.8) [1.3] [C4H7]+
**18c/d***	Coumaroyl-norsolamine	C20H33N3O2	1.95/2.77	348.2639	348.2646	174.6358	174.6359	10	348.2640 (100) [-1.6]	303.2050 (1.4) [-5.6]	-	263.1741 (0.2) [-4.9]	218.1159 (0.5) [-7.6]	202.2277 (0.9) [-0.3]	-	126.1264 (0.3) [-10.5]	100.1122 (5.4) [1.2]	86.0960 (1.6) [-5.0]	-	-	147.0432 (2.0) [-5.8]	119.0479 (0.2) [-10.4] [Acyl-CO]+
								25	348.2632 (9.1) [-3.9]	303.2050 (5.2) [-5.6]	272.1639 (0.5) [-2.2]	263.1734 (0.9) [-7.6]	218.1179 (7.6) [1.6]	202.2273 (5.0) [-2.3]	157.1695 (3.3) [-2.7]	126.1273 (12.9) [-3.4]	100.1121 (100) [0.2]	86.0962 (42.7) [-2.7]	84.0805 (1.2) [-3.3]	58.0645 (1.5) [-10.8]	147.0440 (90.1) [-0.4]	119.0488 (2.5) [-2.9] [Acyl-CO]+, 91.0536 (1.4) [-6.9] [Acyl-2CO]+
								40	-	-	-	-	218.1171 (0.5) [-2.1]	-	-	126.1272 (8.3) [-4.2]	100.1122 (54.0) [1.2]	86.0964 (35.5) [-0.3]	84.0799 (2.6) [-10.5]	58.0651 (4.6) [-0.5]	147.0441 (100) [0.3]	119.0489 (41.8) [-2.0] [Acyl-CO]+, 91.0542 (10.3) [-0.3] [Acyl-2CO]+
**19c/d***	Caffeoyl-norsolamine	C20H33N3O3	1.29/2.24	364.2593	364.2595	182.6338	182.6334	10	364.2600 (100) [1.5]	319.1994 (0.7) [-7.0]	-	279.1705 (0.1) [0.6]	234.1123 (0.3) [-0.7]	202.2261 (0.6) [-8.3]	157.1699 (0.1) [-0.2]	126.127 (0.3) [-5.8]	100.1123 (4.4) [2.2]	86.0963 (1.3) [-1.5]	-	-	163.0383 (1.0) [-4.1]	-
								25	364.2579 (12.1) [-4.3]	319.1998 (3.8) [-5.7]	288.1569 (0.3) [-8.7]	279.1688 (0.7) [-5.4]	234.1116 (7.4) [-3.7]	202.2275 (4.4) [-1.3]	157.1698 (3.2) [-0.8]	126.1275 (10.7) [-1.8]	100.1124 (100) [3.2]	86.0964 (40.8) [-0.3]	84.0808 (0.9) [0.2]	58.0641 (0.7) [-17.7]	163.0389 (61.1) [-0.4]	145.0283 (3.0) [-0.8] [Acyl-H2O]+, 135.0443 (1.6) [1.8] [Acyl-CO]+, 117.0333 (0.9) [-1.6] [Acyl-CO-H2O]+
								40	-	-	-	-	234.1109 (0.8) [-6.7]	-	-	126.1273 (12.8) [-3.4]	100.1124 (86.5) [3.2]	86.0965 (50.7) [0.8]	84.0805 (3.1) [-3.3]	58.0648 (4.9) [-5.7]	163.0391 (100) [0.8]	145.0281 (28) [-2.1] [Acyl-H2O]+, 135.0440 (24.0) [-0.4] [Acyl-CO]+, 117.0335 (14.1) [0.1] [Acyl-CO-H2O]+, 107.0489 (2.9) [-2.2] [Acyl-2CO]+
**20c/d***	Feruloyl-norsolamine	C21H35N3O3	2.45/3.06	378.2754	378.2751	189.6414	189.6412	10	378.2745 (100) [-1.6]	333.2144 (0.9) [-8.6]	-	293.1863 (0.1) [1.1]	248.1267 (0.3) [-5.7]	202.2268 (0.7) [-4.8]	157.1677 (0.3) [-14.2]	-	100.1119 (3.9) [-1.8]	86.0964 (0.7) [-0.3]	-	-	177.0542 (2.0) [-2.4]	-
								25	378.2731 (13.9) [-5.3]	333.2148 (4.3) [-7.4]	-	293.1847 (0.6) [-4.3]	248.1273 (4.3) [-3.3]	202.2262 (7.0) [-7.8]	157.1699 (2.8) [-0.2]	126.1271 (6.5) [-5.0]	100.1117 (78.6) [-3.8]	86.0962 (23.7) [-2.7]	84.0807 (1.1) [-1.0]	58.0641 (1.0) [-17.7]	177.0539 (100) [-4.1]	149.0595 (0.8) [-1.4] [Acyl-CO]+, 145.0284 (10.6) [-0.1] [Acyl-CH3OH]+, 117.0327 (2.2) [-6.8] [Acyl-CO-CH3OH]+
								40	-	-	-	-	248.1277 (0.5) [-1.7]	-	-	126.1275 (11.5) [-1.8]	100.1119 (94.0) [-1.8]	86.0964 (28.3) [-0.3]	84.0806 (3.3) [-2.1]	58.0647 (7.1) [-7.4]	177.0542 (56.5) [-2.4]	149.0586 (9.3) [-7.4] [Acyl-CO]+, 145.0283 (100) [-0.8] [Acyl-CH3OH]+, 134.0362 (1.2) [-0.2] [Acyl-CO-CH3]*+, 117.0331 (24.5) [-3.3] [Acyl-CO-CH3OH]+

* Pairs of E/Z isomers with similar CID mass spectra. Reported mass spectral data were obtained from the later eluting isomer.

Table S6. Analytical data of fatty acyl dinor-solamine **10e**.

no.	name	elemental composition	*ret. time*	$[M+H]^+$ det	$[M+H]^+$ calc	$[M+H]^{2+}$ det	$[M+H]^{2+}$ calc	*CE*	CID mass spectrum: *m/z* (relative intensity in %) [mass error in ppm] — *precursor ion*													
			[min]	[m/z]	[m/z]	[m/z]	[m/z]	[V]	$[M+H]^+$	a^+	b^+	f^+	g^+	c^+	d^+	e^+	Me_2Py^+	$[Py+H]^+$	$[e-C_3H_6]^+$	$[Me_2Py-C_3H_6]^+$	$Acyl^+$	other ions
**10e**	C10:1-dinor-solamine	C20H41N3O	6.19	340.3325	340.3322	170.6702	170.6698	10	340.3332 (100) [2.8]	295.2734 (0.7) [-3.4]	-	-	224.2001 (0.5) [-3.5]	188.2116 (0.3) [-2.8]	-	126.1273 (0.2) [-3.4]	100.1123 (3.4) [2.2]	72.0808 (0.7) [0.3]	-	-	-	-
								25	340.3316 (17.0) [-1.9]	295.2736 (6.2) [-2.7]	278.2468 (1.7) [-3.7]	269.2570 (0.8) [-6.5]	224.2011 (17.4) [0.9]	188.2117 (1.4) [-2.2]	143.1544 (2.1) [0.8]	126.1276 (5.6) [-1.0]	100.1127 (100) [6.2]	72.0809 (24.8) [1.7]	84.0808 (1.5) [0.2]	58.0647 (1.4) [-7.4]	-	135.1160 (1.0) [-6.1] [Acyl-H2O]+, 69.0697 (0.8) [-2.6] [C5H9]+
								40	-	-	-	-	224.1997 (1.4) [-5.3]	-	-	126.1273 (8.2) [-3.4]	100.1125 (100) [4.2]	72.0808 (39.3) [0.3]	84.0805 (6.7) [-3.3]	58.0648 (9.4) [-5.7]	-	135.1161 (1.7) [-5.4] [Acyl-H2O]+, 83.0853 (1.5) [-2.8] [C6H11]+, 69.0695 (8.2) [-5.5] [C5H9]+, 55.0539 (5.1) [-6.0] [C4H7]+
